# Distinct characteristics of T cell receptor repertoire associated with the SARS-CoV-2 reinfection

**DOI:** 10.3389/fimmu.2025.1680089

**Published:** 2025-10-23

**Authors:** Liling Zeng, Li Liu, Baolin Ren, Bing Feng, Xudong Lai, Xunxi Lai, Zhimin Chen, Yihui Huang, Wenxin Hong

**Affiliations:** ^1^ The Second Affiliated Hospital of Guangzhou University of Chinese Medicine (Guangdong Provincial Hospital of Chinese Medicine), The Second Clinical Medical College of Guangzhou University of Chinese Medicine, Guangzhou, China; ^2^ Guangzhou Red Cross Hospital, Jinan University, Guangzhou, China; ^3^ Guangdong Polytechnic Normal University, Guangzhou, China; ^4^ Guangzhou Eighth People’s Hospital, Guangzhou Medical University, Guangzhou, China

**Keywords:** SARS-CoV-2 reinfection, TCR repertoire, immune protection, clonal expansion, V(D)J usage

## Abstract

The COVID-19 pandemic, caused by SARS-CoV-2, represents one of the most profound global public health challenges in modern history. While T cell immunity is crucial for viral clearance, the dynamics of the T cell receptor (TCR) repertoire during reinfection remain poorly understood. This study sought to characterize the TCR repertoire in peripheral blood T cells from healthy convalescent individuals (HC), patients with primary SARS-CoV-2 infection (PI), and reinfected individuals (RI), aiming to identify distinct TCR signatures linked to susceptibility or protection against reinfection. We enrolled 48 age- and sex-matched participants (18 PI, 18 RI, 12 HC), collecting blood samples during acute infection (PI/RI) or convalescence (HC). Deep TCRα/β sequencing was performed using the SMARTer Human TCR Profiling Kit with unique molecular identifiers (UMIs), followed by analysis of TCR repertoire diversity, clonal expansion, V(D)J gene usage, and CDR3 characteristics. Compared to HC, both PI and RI groups exhibited significantly reduced TCR diversity (*p*< 0.001), though no significant differences were observed between PI and RI. COVID-19 patients displayed skewed TCR repertoires dominated by expanded clones (>1%), whereas HC primarily harbored small clones (≤ 0.1%). RI patients demonstrated intermediate clonality, suggesting partial memory recall. Group-specific V(D)J pairings were identified, including TRAV27/TRAJ42 in RI, TRAV24/TRAJ42 in PI, and TRAV35/TRAJ42 in HC, while TRBV6-4/TRBD2/TRBJ2–3 was conserved across all groups. Additionally, HC-enriched and RI-exclusive CDR3 clusters were detected. Our findings indicate that SARS-CoV-2 reinfection is associated with impaired TCR diversity and distinct clonal expansion patterns, underscoring the role of T cell immunity in reinfection susceptibility. HC-enriched TCR clusters may represent protective memory responses, whereas RI-specific signatures suggest compromised immunity. These results offer valuable insights for vaccine design and risk stratification, though further functional validation of the identified TCRs is necessary.

## 1 Introduction

The COVID-19 pandemic, caused by severe acute respiratory syndrome coronavirus 2 (SARS-CoV-2), represents one of the most profound global public health crises in modern history (1). Emerging cases of reinfection, defined as recurrent PCR positivity occurring ≥90 days after initial infection (or within 90 days with symptom-free intervals supported by two negative tests) (2), have raised renewed concerns. Global reinfection rates vary widely, ranging from 0.77% to 12.7% (3–6), with key risk factors including viral evolution (7, 8), advanced age, comorbidities, and particularly waning immunity (9–11).

Research indicates that COVID-19 patients exhibit significantly reduced peripheral blood T-lymphocyte counts, a finding clinically associated with disease severity (12). This lymphopenia may contribute to compromised immune responsiveness. Critically, a single prior infection has been shown to undermine subsequent T-cell responses, as evidenced by diminished CD8^+^ T-cell activation and expansion following vaccination in convalescent individuals (13). This establishes that even one infection can compromise the immune system’s capacity to respond to new challenges, raising the concern that repeated infections may have cumulative detrimental effects. Reinfection causes exacerbated long-term sequelae, including immune dysfunction and cardiovascular complications, and leads to an increased all-cause mortality risk (11, 14). Significantly, the typical 4~6 month interval between initial infection and reinfection (11), suggesting humoral immune deficiency alone cannot fully explain reinfection susceptibility, a conclusion consistent with prior research (15). We therefore hypothesize that T-cell immune deficiency critically underpins susceptibility to reinfection.

T cell-mediated immunity plays a dual role in COVID-19 pathogenesis, contributing to both viral clearance and pathological inflammation. Mounting evidence indicates SARS-CoV-2-specific T cells are essential for durable protection, with initial infection severity inversely correlating with reinfection risk (16), a relationship attributable to the quality and persistence of memory T-cell responses. Consequently, the T-cell receptor (TCR) repertoire, generated through V(D)J recombination of α/β chains, serves as a molecular fingerprint of antigen-specific immunity (17). While TCR diversity metrics and clonal expansion patterns provide critical insights into immune dynamics, features distinguishing protective from insufficient responses remain incompletely characterized, particularly in reinfection contexts. Public TCR clonotypes recognizing immunodominant epitopes are frequently shared across individuals (18), whereas private repertoires may influence individual susceptibility. Although SARS-CoV-2-specific TCRs have been identified via single-cell sequencing (with sequences cataloged in VDJdb) and linked to clinical outcomes (19–21), no systematic comparison exists across three critical cohorts: primary infection, reinfection, and protected convalescent individuals resisting reinfection. To address this gap, we performed deep TCR α/β sequencing on peripheral blood from these three rigorously phenotyped cohorts to identify TCR signatures that distinguish susceptibility to reinfection from protective immunity and elucidate mechanisms underlying sustained T-cell-mediated immunity.

## 2 Materials and methods

### 2.1 Study participants and sample collection

All participants were aged 20–40 years, representing the demographic with higher reinfection risk (22). Exclusion criteria included: immunosuppressive therapy, HIV infection, active cancer treatment.

Peripheral blood samples (8 mL) were collected by venipuncture using PAXgene Blood RNA tubes (PreAnalytiX, Hombrechtikon, Switzerland) containing proprietary RNA stabilization reagents. For the acute infection groups (PI and RI), samples were obtained within 96 hours (4 days) of symptom onset to capture peak adaptive immune responses (22). Convalescent samples (HC) were collected ≥9 months post-recovery to ensure resolution of acute infection. Immediately after collection, samples were gently inverted 8–10 times to ensure proper mixing with stabilization buffer, then maintained at room temperature (20-25 °C) for exactly 4 hours to allow complete RNA stabilization before storage at -80 °C.

### 2.2 RNA extraction and quality control

Total RNA was extracted from samples using the PAXgene Blood RNA Kit (QIAGEN, Hilden, Germany) in accordance with the manufacturer’s instructions. RNA concentration was measured using the Agilent 2100 Bioanalyzer with RNA Nano chips (Agilent Technologies, Santa Clara, CA), with a minimum acceptable concentration of ≥20 ng/μL. RNA integrity was evaluated using the Qsep-400 system based on the following criteria: 28S/18S (or 23S/16S) ribosomal RNA ratio ≥1.0; RNA Integrity Number (RIN) ≥7.0.

### 2.3 TCR α/β repertoire profiling and analysis

#### 2.3.1 TCR library preparation

TCR repertoire profiling was performed using the SMARTer Human TCR α/β Profiling Kit v2 (Takara Bio USA, Inc., Mountain View, CA), which incorporates the following key technologies: first, SMART (Switching Mechanism at 5’ End of RNA Template) technology was employed using a 5’ RACE-based approach to capture complete V(D)J variable regions from TCR transcripts while incorporating unique molecular identifiers (UMIs) for clonotype quantification. Second, first-strand cDNA synthesis was initiated by TCR-specific oligo-dT priming using MMLV-derived SMARTScribe Reverse Transcriptase. This enzyme adds 3–5 nontemplated nucleotides upon reaching mRNA 5’ ends, enabling subsequent annealing of the TCR SMART UMI Oligo (containing 12-nt random sequences) during template switching. The incorporated universal sequence served as primer binding sites for PCR amplification. Third, library quality control measures. Final libraries were validated for successful production, purification, and size selection using the Agilent 2100 Bioanalyzer (High Sensitivity DNA chip).

#### 2.3.2 TCR α/β sequencing and data preprocessing

The SMARTer TCR α/β Profiling Kit is optimized for use with Takara Bio’s Cogent™ NGS Immune Profiler Software, which improves clonotype calling accuracy by leveraging UMIs to eliminate PCR duplicates and errors. Single-stranded library products are generated through denaturation, followed by circularization to form single-stranded DNA circles. Residual linear DNA molecules are enzymatically digested to ensure purity. The circularized library is then amplified via phi29-mediated rolling circle amplification (RCA), producing DNA nanoballs (DNBs) containing over 300 copies of each original molecule. These DNBs are deposited onto a patterned nanoarray and sequenced using paired-end 300 bp (PE300) reads on the BGI G400 platform (BGI-Shenzhen, China).

Raw sequencing data (FASTQ files) from the BGISEQ-2000 were processed using MiXCR. Low-quality reads were filtered out, including: Reads with poly-N sequences or missing adapters/insert tags; Reads with adapter contamination or poly-A/T/G/C artifacts; Reads shorter than 200 bp, containing 6-bp homopolymers, or with primer mismatches; Reads with a Phred quality score (Q-score) below 19. Additionally, Q20, Q30, and GC content metrics were calculated to assess data quality. The resulting clean reads were used for downstream analysis.

#### 2.3.3 TCR α/β repertoire analysis

After repertoire size normalization, TCR repertoire analysis began with the alignment of filtered reads to V, D, J, and C gene segments of the TCR alpha (TRA) and beta (TRB) loci using MiXCR for clonotype assembly. The core output from VDJtools included clonotype counts, frequencies, CDR3(complementarity-determining region 3) nucleotide sequences, CDR3 amino acid (complementarity-determining region 3 amino acid, CDR3AA) sequence, and gene segment boundaries (V end, D start/end, J start). Diversity metrics (Chao1, ChaoE, Shannon index, Inverse Simpson) quantified repertoire heterogeneity. Clonality was categorized based on frequency: small clones (≤ 0.1%); medium clones (0.1–1%); expanded clones (>1%). To facilitate downstream analyses, alignment results were annotated with critical features, including clone counts, clone frequencies, V/D/J gene alignments, and CDR3AA sequences.

Dominant V(D)J rearrangement patterns were identified by aggregating data across samples. For high-frequency V(D)J combinations, and amino acid (CDR3aa) sequences were analyzed to pinpoint recurrent CDR3 motifs. Additional analyses included: V(D)J gene usage: Top gene segments and COVID-19-associated sequences (matched via VDJdb); CDR3 characterization: Length distribution, motif enrichment (MEME Suite), and temporal trends (Mfuzz clustering).

#### 2.3.4 SARS-CoV-2-specific TCR analysis

COVID-19-associated clonotypes were identified by screening the total TCR repertoire against known SARS-CoV-2-specific CDR3β amino acid (AA) sequences from the VDJdb database, a curated repository of experimentally validated T-cell receptor-antigen interactions. To identify SARS-CoV-2-reactive TCRs in our dataset, we performed exact CDR3β (or CDR3α) amino acid sequence matching between the VDJdb-derived SARS-CoV-2-Specific TCR sequences and our experimentally detected TCR repertoires. Using this approach, we analyzed: V, D, and J gene segment usage among SARS-CoV-2-associated TCRs; CDR3AA (complementarity-determining region 3 amino acid) sequence features to characterize SARS-CoV-2-specific T-cell immune responses. This enabled the identification of potential COVID-19-related TCR clonotypes and provided insights into the adaptive immune response to SARS-CoV-2.

### 2.4 Statistical analyses

All statistical analysis was implemented with R software (Version 4.2.3, downloaded from http://www.r-project.org, accessed on 8 January 2024). All multi-group comparisons were performed using the Kruskal–Wallis test, followed by Dunn’s *post-hoc* test for non-normally distributed data. TCR diversity was visualized using boxplots. SARS-CoV-2-specific clonotypes were clustered based on CDR3AA frequencies using the Mfuzz method (clusterGvis R package, v0.1.2). For motif enrichment analysis, the MEME suite (v5.5.7) was employed with default parameters. Frequency heatmaps were generated using the pheatmap package (v1.0.12), and Venn diagrams were constructed with ggvenn (v0.1.10). Additional visualizations, including bar plots and violin plots, were created using ggplot2.

## 3 Results

### 3.1 Participant recruitment

**Figure 1:**
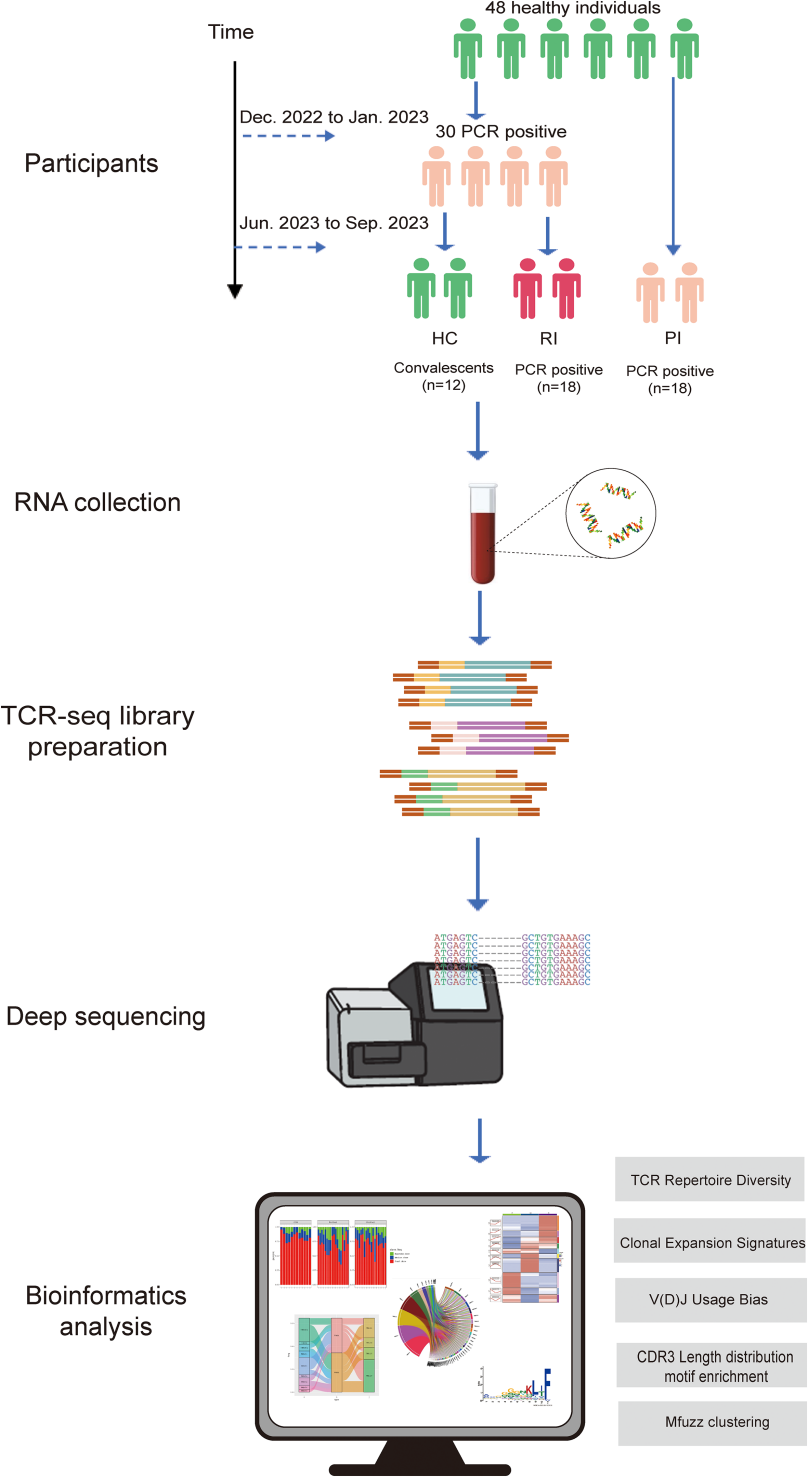
Research workflow diagram. PCR positive, SARS-CoV-2 infection confirmed by reverse transcription polymerase chain reaction method; HC, healthy convalescent group; RI, reinfection group; PI, primary infection group.

Study participants were recruited from the Guangzhou Red Cross Hospital in Guangdong Province, China, between December, 2022, and September, 2023. A total of 48 participants across three cohorts were enrolled for deep TCRα/β sequencing (see **Figure 1**). Primary infection (PI) group (n=18): Individuals with their first SARS-CoV-2 infection during China’s second pandemic wave (after April 2023), confirmed by Reverse Transcription Polymerase Chain Reaction (RT-PCR) and symptomatic (by WHO criteria), and no prior exposure history; Reinfection (RI) group (n=18): Patients with RT-PCR-confirmed infections during both the initial wave (December 2022) and the second wave. Reinfection, defined as recurrent PCR positivity occurring ≥90 days after initial infection (or within 90 days with symptom-free intervals supported by two negative tests); Healthy convalescent (HC) group: comprised 12 healthcare workers infected for the first time during the initial relaxation of the ‘Zero-COVID’ policy, with no reinfections observed over a nine-month follow-up.

### 3.2 Clinical and demographic data

**Figure 2:**
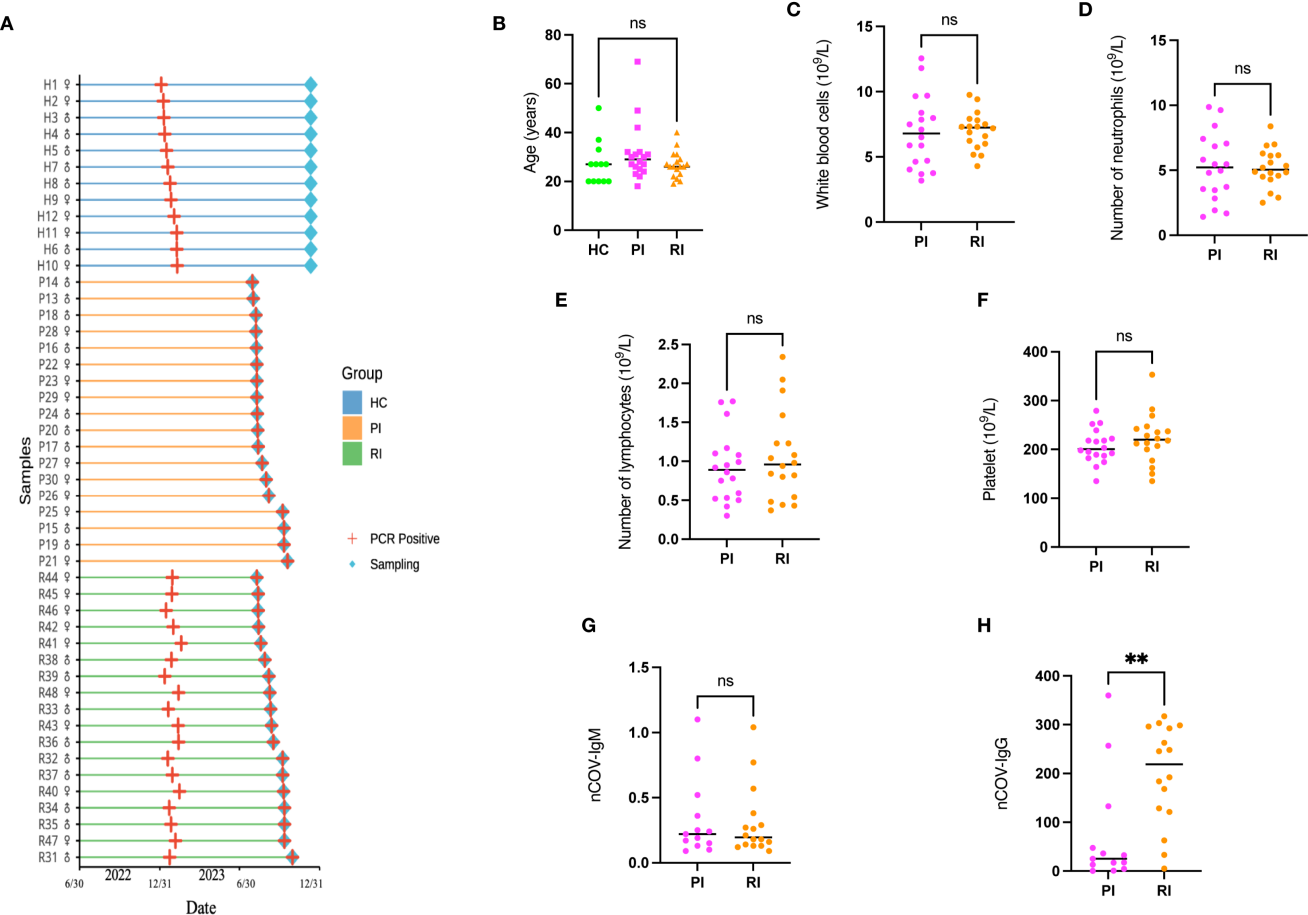
Study population flowchart and characteristics. **(A)** A total of 36 SARS-CoV-2-positive subjects (18 with primary infection [PI], 18 with reinfection [RI]; gender ratio 1:1) and 12 healthy controls (HC; gender ratio 1:1) were enrolled. Peripheral blood samples were collected for T-cell receptor (TCR) sequencing. **(B)** Age distribution of participants compared using a one-way ANOVA. **(C–F)**. Routine inflammatory markers in SARS-CoV-2-infected patients. **(G–H)**. SARS-CoV-2-specific antibody distribution. Data represent mean ± SD. Statistical significance was determined by two-tailed *t*-test: **P < 0.01, ns, not significant..

To assess blood immune profiles across COVID-19 disease stages, this study enrolled 48 age- and sex-matched participants (1:1 male-to-female ratio) from Guangzhou Red Cross Hospital, including 12 HC, 18 PI, and 18 RI (**Figure 2A**, detailed in **Supplementary Table 1**). The HC group was infected for the first time, with no reinfections observed during the nine-month follow-up period. The PI group was infected during the second wave without prior exposure, while the RI group experienced infection during both the first and second waves (**Figure 2A**). Pandemic progression during these periods is detailed in **Supplementary Figure 1**.

The cohort comprised individuals aged 20~40 years, representing the demographic most susceptible to reinfection (23), all of whom manifested mild clinical symptoms. No significant differences were observed in age distribution, routine inflammatory markers (white blood cell, neutrophil, lymphocyte, or platelet counts) or SARS-CoV-2-specific IgM levels between PI and RI groups (detailed in **Supplementary Table 1**). However, IgG titers were significantly elevated in the RI group compared to the PI group *(p* < 0.001) (**Figure 2**).

### 3.3 TCR repertoire diversity loss in acute infection

Peripheral blood samples were collected from all participants for RNA isolation. TCRαβ repertoires were profiled using a 5’ RACE-like approach across HC, PI, and RI groups. None of the subjects had received anti-infective or immunomodulatory therapies for at least six months prior to sampling.

**Figure 3:**
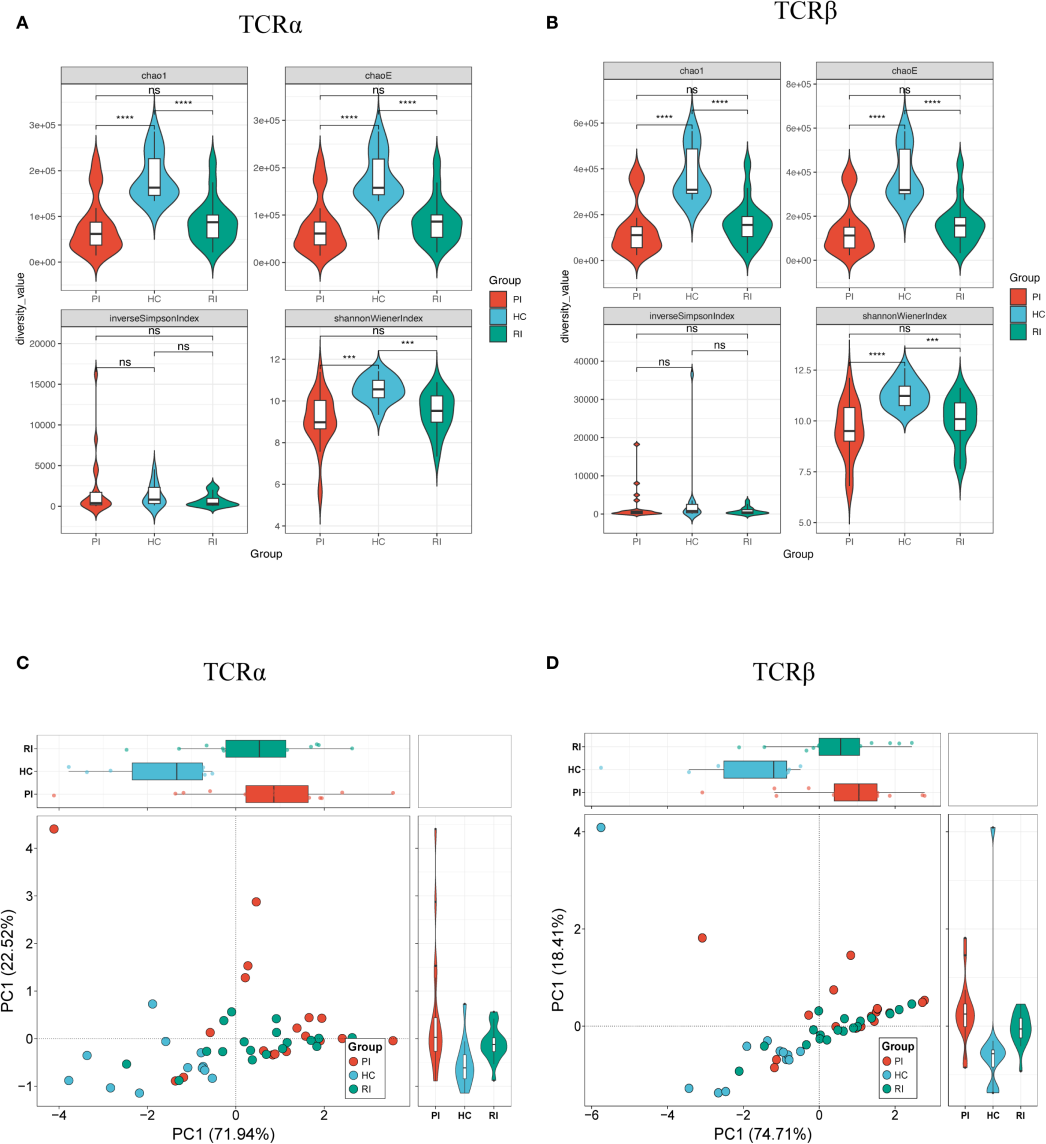
Diversity analysis of TCRαβ repertoires. Repertoire diversity was assessed using four metrics: Chao1, ChaoE, Inverse Simpson index, and Shannon index. **(A)** TCRα chain diversity; **(B)** TCRβ chain diversity; **(C)** Principal component analysis (PCA) of TCRα diversity; **(D)** PCA of TCRβ diversity. Data represent mean ± SD. Statistical significance was determined by Kruskal-Wallis test with Dunn’s *post-hoc* test: ***P < 0.001, ****P < 0.0001. ns, not significant.

Diversity indices (Chao1, ChaoE, Shannon index, and Inverse Simpson index) were calculated to evaluate TCR repertoire heterogeneity (**Figures 3A, B**; **Supplementary Figure 2**). Higher values indicate greater diversity. Compared to HC, COVID-19 patients (both PI and RI groups) exhibited significantly reduced TCR clonal diversity, with lower Chao1, Shannon indices, and Inverse Simpson index (*p* < 0.001; **Figures 3A, B**). Notably, no significant diversity differences were observed between PI and RI groups, suggesting similar TCR repertoire impairment during primary infection and reinfection.

To evaluate inter-group similarity, principal component analysis (PCA) was performed using all four diversity indices (ChaoE, Chao1, Shannon-Wiener index, and Inverse Simpson index) as variables. PCA revealed clear separation between healthy controls and COVID-19 patients (**Figures 3C, D**), further corroborating the diversity differences.

### 3.4 Clonal expansion signatures

**Figure 4:**
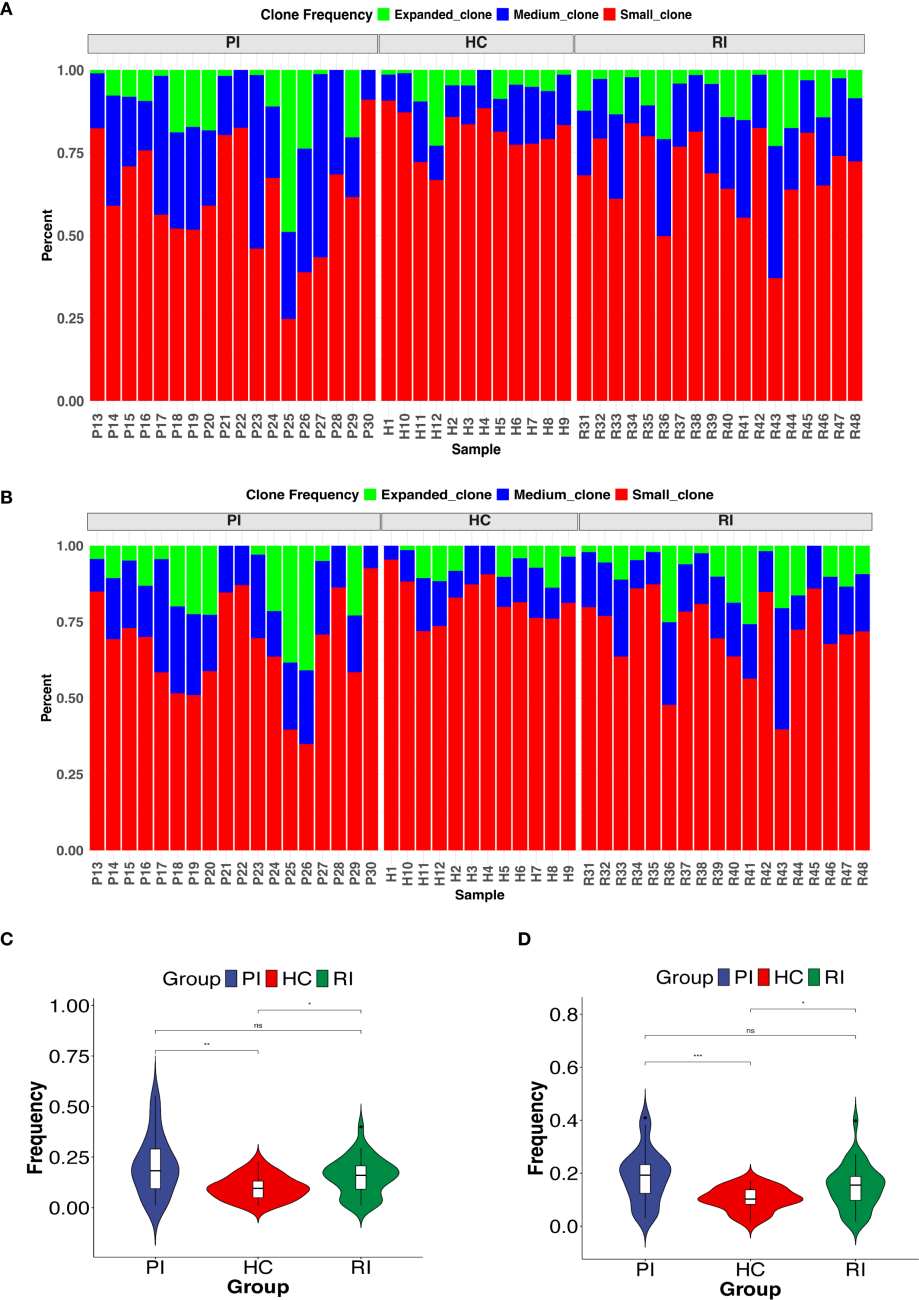
Clonal expansion of the T-cell repertoire across groups. **(A ,B)** Distribution of TCR clonotypes by size: small (0 < x ≤ 0.1%), medium (0.1% < x ≤ 1%), and expanded (x > 1%). **(C, D)** Comparison of highly expanded clonotypes between groups. *P < 0.05, **P < 0.01, ***P < 0.001. ns, not significant.

Next, we compared TCRαβ clonal expansion across groups. Upon activation, T cells undergo clonal expansion, a process in which antigen-stimulated T cells proliferate rapidly, generating large clones with identical TCRs. To evaluate clonal expansion, we analyzed the frequency distribution of TCR clonotypes within the repertoire. Based on their frequencies, TCR clonotypes were categorized into three groups: expanded (>1%), medium (0.1%–1%), and small (≤0.1%). Compared to healthy controls (HC), COVID-19 patients exhibited a significantly skewed TCR repertoire, with a higher proportion of highly expanded clonotypes (**Figures 4A, B**). Further analysis revealed that RI patients had a greater proportion of clonally expanded TCRs than HC but showed a lower trend than the PI group. This RI-specific pattern, marked by intermediate clonality, may reflect partial memory recall (**Figures 4A, B**). Overall, both PI and RI COVID-19 patients displayed higher clonality than HC (*P* < 0.05) (**Figures 4C, D**).

Additionally, we examined the frequency distribution of COVID-19-related TCR clonotypes across groups. Most HC subjects predominantly harbored small TCR clones, whereas nearly half of COVID-19 patients exhibited medium-sized clones (**Supplementary Figure 3**). These findings indicate that COVID-19 patients possess a distinct T-cell clonality profile compared to HC, with varying degrees of TCR repertoire reuse among the three groups.

### 3.5 V(D)J usage bias

We captured complete V(D)J variable regions of TCR transcripts from peripheral blood samples. T cell receptors are generated through the rearrangement of V and J gene segments for the TCRα chain and V, D, and J gene segments for the TCRβ chain.

From the peripheral blood samples, we obtained a total of 204,461,742 VJ gene combinations for TCRα (HC: 49,339,581; PI: 74,914,332; RI: 80,207,829) and 9,875,062 VDJ gene combinations for TCRβ (HC: 2,457,157; PI: 4,539,433; RI: 2,878,472) (**Supplementary Figure 4**). Next, we matched the COVID-19-associated V, D, and J gene segments from the VDJ database with the V-J pairings or V-D-J pairings of the TCRα (V-J) and TCRβ (V-D-J) pairings in our dataset to identify COVID-19-related VDJ combinations in each group. This analysis identified 139,944 TCRα VJ combinations (HC: 57,399; PI: 37,626; RI: 44,919; accounting for 0.07% of total TCRα VJ gene combinations) and 40,321 TCRβ VDJ combinations (HC: 18,128; PI: 10,291; RI: 11,902; accounting for 0.41% of total TCRβ VDJ gene combinations) (**Supplementary Figure 4**).

**Figure 5:**
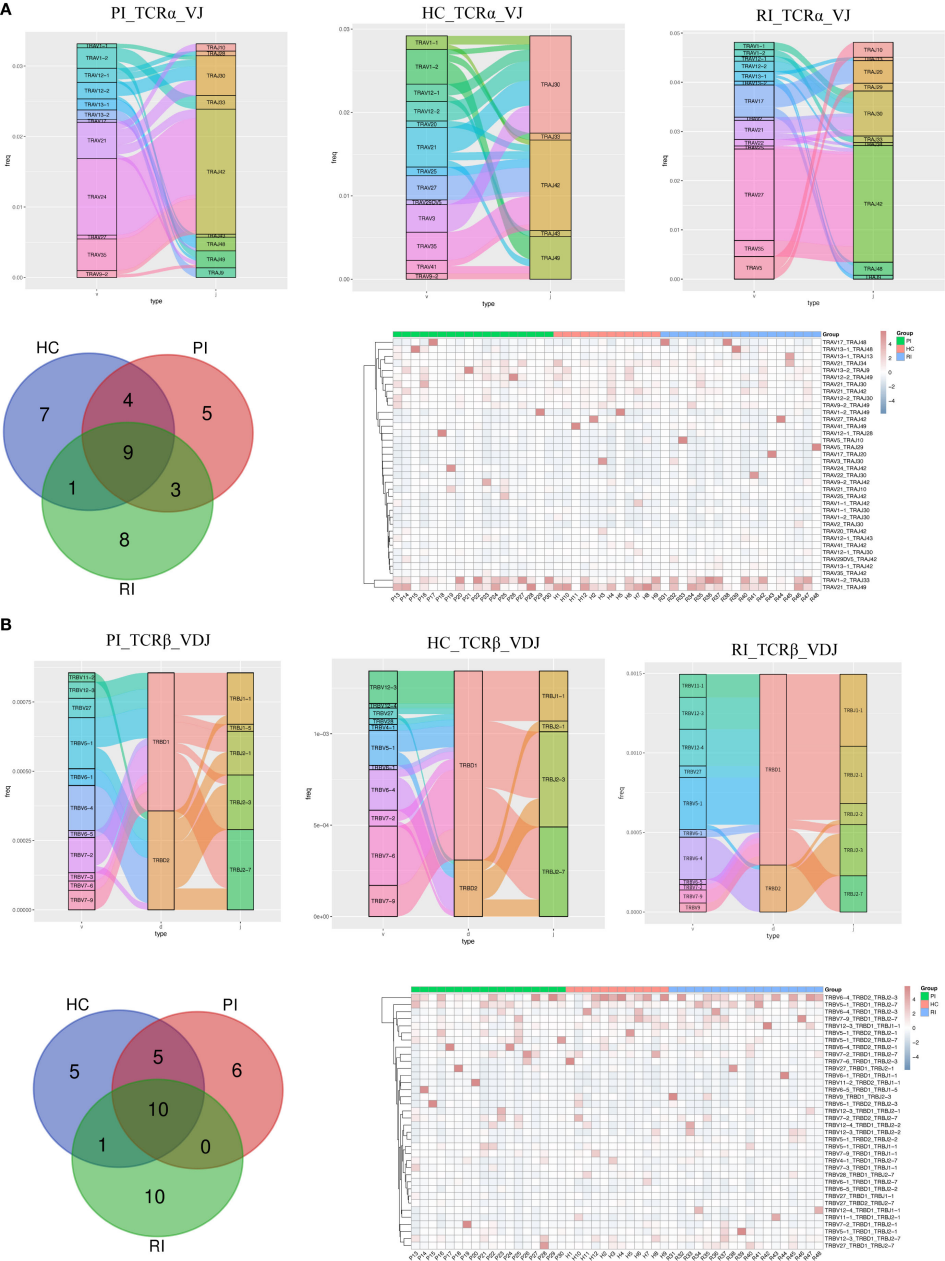
Comparison of V, D, and J gene usage in TCRα (TRA) and TCRβ (TRB) chains. **(A)** Sankey diagram, Venn diagram and heatmaps showing the top 20 VJ pairings of TCRα across groups. **(B)** Sankey diagram, Venn diagram, and heatmaps displaying the top 20 VJ pairings of TCRβ across groups.

First, we analyzed the overall usage of V, D, and J gene segments in TCRα and TCRβ and observed that the most frequently used segments varied among groups (**Supplementary Figure 5**). **Figure 5** displays the top 20 COVID-19-associated VDJ patterns for TCRα and TCRβ. In PI subjects, the most frequently used segments were TRAV24/TRAJ42 for TCRα and TRBV6-4/TRBD2/TRBJ2–3 for TCRβ; In HC subjects, the dominant segments were TRAV35/TRAJ42 for TCRα and TRBV7-6/TRBD1/TRBJ2–3 for TCRβ; In RI subjects, the predominant segments were TRAV27/TRAJ42 for TCRα and TRBV6-4/TRBD2/TRBJ2–3 for TCRβ (**Figures 5A, B**; **Supplementary Figure 6**).

To determine whether unique V(D)J recombination patterns are specific to COVID-19 patients, we further compared the top 20 V(D)J pairings in individual subjects. Venn diagrams (**Figure 5**) show that PI subjects exhibited 5 unique TRA VJ pairs (e.g., TRAV12-2/TRAJ49) and 6 unique TRB VDJ pairs (e.g., TRBV7-2/TRBD1/TRBJ2-1); HC showed 7 unique TRA VJ pairs (e.g., TRAV1-1/TRAJ42) and 5 unique TRB VDJ pairs (e.g., TRBV27/TRBD2/TRBJ2-7); RI subjects displayed 8 unique TRA VJ pairs (e.g., TRAV5/TRAJ10) and 10 unique TRB VDJ pairs (e.g., TRBV11-1/TRBD1/TRBJ2-1). The details of the top unique V(D)J pairs per cohort are provided in **Supplementary Table 2**.

Notably, the heatmaps (**Figure 5**) show all top 20 paired TCRαβ clonotypes. Several common TRA and TRB pairs, such as TRAV1-2/TRAJ33, TRAV21/TRAJ49, TRBV6-4/TRBD2/TRBJ2-3, and TRBV5-1/TRBD1/TRBJ2-7, were consistently detected from the primary infection (PI) through the convalescent phase (HC) and into the reinfection phase (RI) (**Supplementary Figure 7**). This persistent presence suggests that T cell clones with these recombination patterns may represent memory T cell phenotypes. These clones likely expanded and contributed to the anti-SARS-CoV-2 immune response across different phases of infection and recovery.

In summary, our findings demonstrate that VJ pairings in TCRα and TCRβ chains differ between COVID-19 patients and healthy controls, highlighting potential immune repertoire signatures associated with SARS-CoV-2 infection.

### 3.6 CDR3 length distribution and motif enrichment

**Figure 6:**
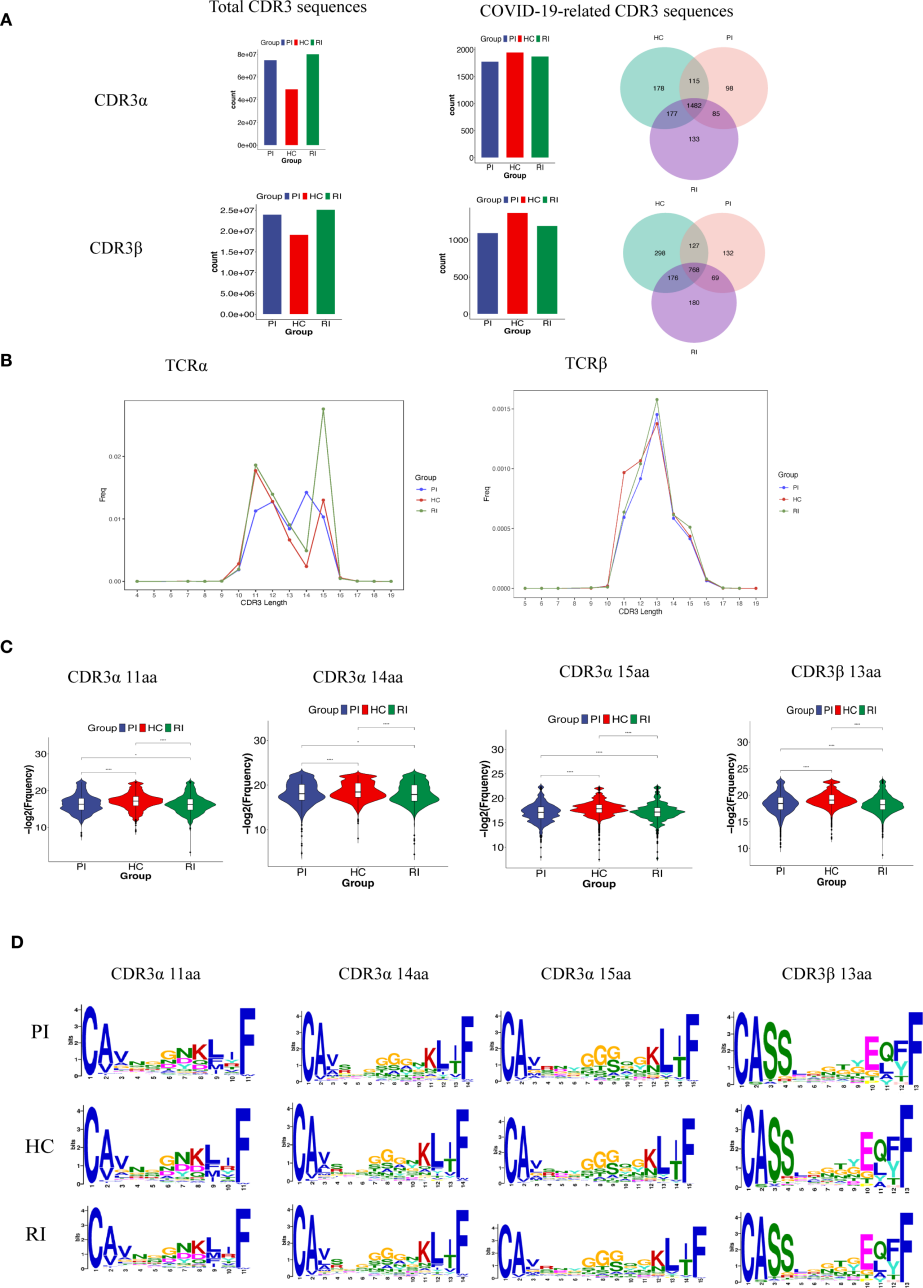
TCRαβ CDR3 repertoire characteristics across patient cohorts. **(A)**. Counts of TCRαβ CDR3 clonotypes in the three patient groups. **(B, C)**. CDR3 length distribution of clonally expanded TCRs. *P < 0.05, ****P < 0.0001; **(D)**. CDR3α and CDR3β motifs enriched by CDR3 length, identified using MEME Suite (v5.5).

Our TCR repertoire analysis yielded 204,461,742 CDR3α sequences (HC: 49,339,581; PI: 74,914,332; RI: 80,207,829) and 68,307,891 CDR3β sequences (HC: 19,156,067; PI: 23,990,022; RI: 25,161,802) (**Figure 6A**). To identify SARS-CoV-2-associated TCRs, COVID-19-associated clonotypes (n = 10,349, as shown in **Appendix Table S1**) were identified by filtering the total TCR repertoire against known SARS-CoV-2-Specific CDR3 amino acid (AA) sequences from the VDJdb database. We matched these sequences with COVID-19-related CDR3α/CDR3β data from the VDJ database. This analysis revealed 5,609 shared TCRα sequences (HC: 1,952; PI: 1,780; RI: 1,877; accounting for 0.003% of total CDR3α sequences) and 3,658 shared TCRβ sequences (HC: 1,369; PI: 1,096; RI: 1,193; accounting for 0.005% of total CDR3β sequences) (**Figure 6A**). Among these, 1,482 (65.3%) TCRα and 768 (43.9%) TCRβ sequences were common across all three groups (as shown in the Venn diagram, **Figure 6A**).

Firstly, we compared the amino acid (AA) length distributions of CDR3α and CDR3β in our experimentally detected TCR repertoires. A modest difference in CDR3β length was observed among groups, while CDR3α lengths showed no significant variation (**Supplementary Figure 8**).

Next, we examined the aa length distribution of COVID-19-associated CDR3 regions. CDR3 lengths were largely consistent across groups, with most sequences falling within the 9~17 aa range. For TCRα, the predominant lengths were 14 aa in the PI group, 11 aa in HC, and 15 aa in RI. In TCRβ, the most frequent length across all groups was 13 aa (**Figure 6A**).

However, for clonally expanded TCRs, the proportion of T cells sharing identical CDR3 lengths differed significantly among groups, with the RI group exhibiting the highest prevalence (**Figures 6B, C**). Enrichment analysis of differentially expanded CDR3 motifs across the three groups demonstrated high similarity among groups repertoires (**Figure 6D**).

### 3.7 Temporal trends (Mfuzz clustering) reveals distinct CDR3

To determine whether dynamic CDR3 sequence patterns are specific to HC or COVID-19 patients (PI/RI), we analyzed the frequency trends of SARS-CoV-2-associated TCRα and TCRβ CDR3 AA sequences across groups.

First, we extracted all COVID-19-associated CDR3AA clones and applied Mfuzz clustering to group sequences with similar frequency dynamics. Trend line graphs and heatmaps visualized these clusters, revealing potential disease-associated signatures (**Supplementary Figure 9**; The CDR3 sequence details for each cluster can be found in **Appendix Tables S2**, **S3**).

**Figure 7:**
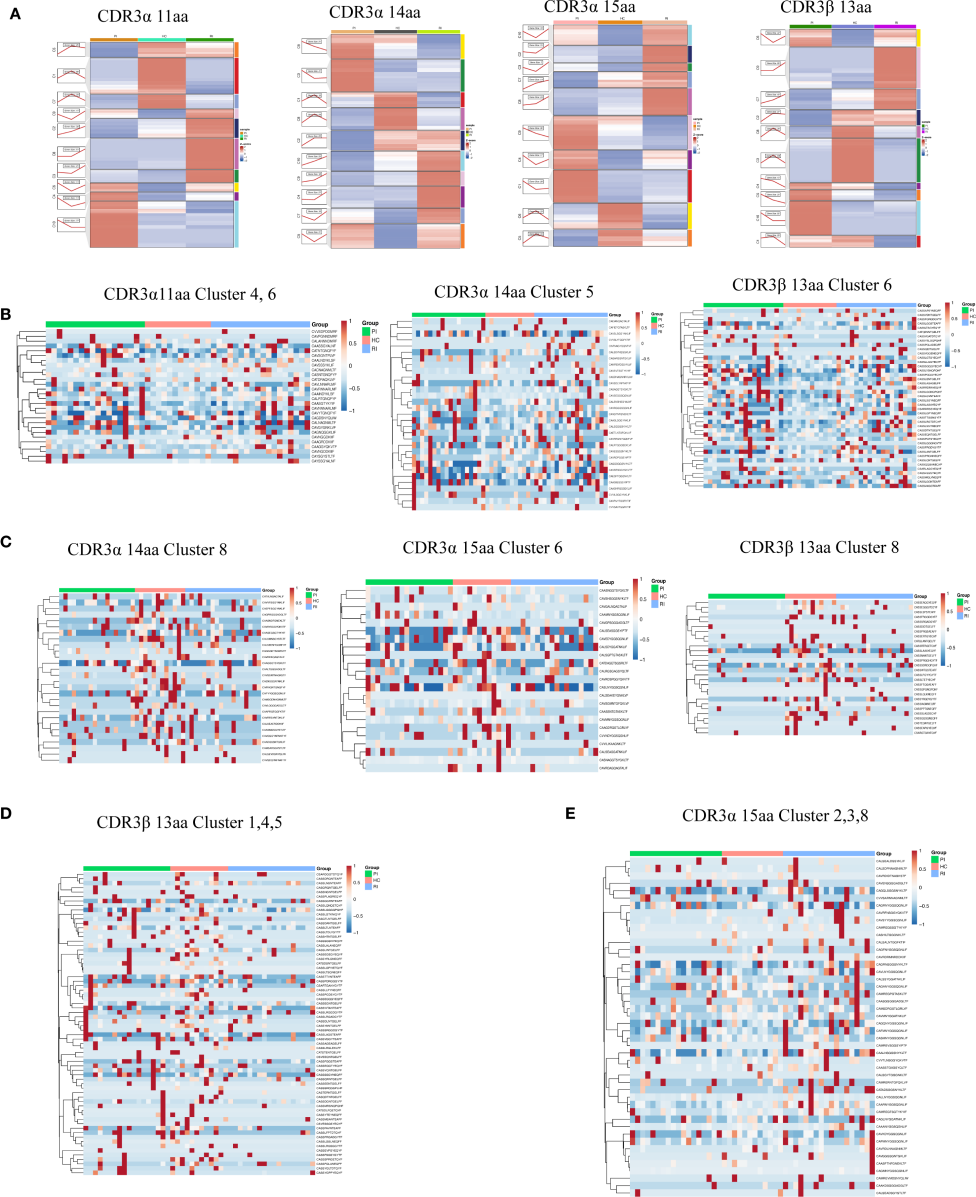
Length-specific clustering of TCRα and TCRβ CDR3 sequences. **(A)** Temporal trends (Mfuzz clustering) across three groups. **(B)** CDR3α (11aa, 14aa) and CDR3β (13aa) clusters associated with HC and low clonal expansion. **(C)** CDR3α (14aa, 15aa) and CDR3β (13aa) clusters associated with HC and high clonal expansion. **(D)** CDR3β (13aa) clusters associated with RI and low clonal expansion. **(E)** CDR3α (15aa) clusters associated with RI and high clonal expansion.

Based on the findings from the previous section, we further focused on TCRα clones with CDR3AA lengths of 11, 14, and 15, and on TCRβ clones with a length of 13. Mfuzz clustering grouped these sequences based on their frequency trends across PI, HC, and RI stages (**Figure 7A**). Although these clones exhibited clonal expansion, their frequency dynamics differed significantly among groups (**Figure 7A**). Heatmaps visualized representative CDR3 sequence motifs with differential lengths in PI, HC, and RI, revealing group-specific signatures (**Figures 7B–E**; detailed CDR3 sequences per cluster are provided in **Appendix Tables S4–S7**). In **Figure 7B**, clones in PI/RI-Dominant Clusters (acute infection or recall signatures) sharply declined from PI to HC but rebounded in RI, suggesting re-expansion of infection-associated TCRs during reinfection. Clones in HC-Enriched Clusters (protective memory T-cell populations) exclusive to HC (**Figure 7C**) were exclusively detected in HC, these clones may represent long-lived memory or regulatory subsets. Their absence or low expansion in PI/RI suggests distinct antigenic pressures during acute infection versus convalescence. While they likely contribute to immune surveillance, their role in reinfection prevention requires further validation (**Figure 7C**). RI-Exclusive Clusters (Reinfection Signatures), which is unique to RI (**Figures 7D, E**), may represent distinct TCR repertoire features and CDR3 sequences associated with SARS-CoV-2 reinfection (The CDR3 sequence details for each cluster can be found in **Appendix Tables S4–S7**).

Notably, most activated T-cell clonotypes recognize viral proteins encoded by ORF1ab, the nucleocapsid, and the spike protein. Comparing HC and RI individuals, we identified 94 shared TCRα sequences (targeting ORF1ab [39], nucleocapsid [19], spike [24]) among all HC patients, far fewer were shared in RI patients despite infection with the same virus. Similarly, 265 TCRβ sequences (ORF1ab [87], nucleocapsid [54], spike [108]) were shared across HC patients but were significantly less prevalent in RI patients (The CDR3 sequence details for each cluster can be found in **Appendix Tables S8, S9**; **Supplementary Figure 10**).

## 4 Discussion

The TCRα/β repertoire shapes epitope-specific T cell responses, influencing immunodominance, functionality, and protective efficacy. Defining precise immune correlates of protection against SARS-CoV-2 reinfection is essential for predicting population-level disease trajectories, guiding public health interventions, and advancing targeted therapeutic strategies. As the first comprehensive analysis of TCR repertoire dynamics across heterogeneous SARS-CoV-2 infection histories, this work provides mechanistic insights into immune protection with direct implications for next-generation vaccine design and clinical risk stratification. Below, we discuss key findings and their implications within the epidemiological and immunological context.

China maintained a dynamic zero-COVID policy for over two years prior to its relaxation in early December 2022 (24, 25). This shift precipitated nationwide surges in symptomatic infections, with 60~80% of urban populations infected during the initial wave (26, 27). While community transmission subsided within 4~6 weeks, reinfection cases emerged by April 2023, triggering a secondary epidemic wave (shown in the **Supplementary Figure 1**). Our sample collection coincided with this reinfection phase.

Notably, reinfections predominantly affected young adults (20 to 40 years old) (23), reflecting both heightened exposure risk and their pivotal role in viral transmission. Accordingly, we prioritized this demographic. Sampling was collected during the acute infection phase (2–4 days post-symptom onset), strategically timed to capture peak viral loads and immune activation as established in challenge studies (22). Despite maximal clonal expansion during this window, SARS-CoV-2-associated TCRs remained exceptionally rare (0.003% of CDR3α and 0.005% of CDR3β sequences; **Figure 6A**), consistent with prior findings (22), reflecting the high diversity of the T-cell response and the compartmentalized nature of antigen-specific clones. Such low prevalence complicates the detection of these activated clonotypes and their distinction from bystander sequences when relying solely on enrichment analysis of the full immune repertoire between healthy and infected individuals. This highlights the key advantage of our novel method in identifying SARS-CoV-2-associated TCR signatures. To identify SARS-CoV-2-reactive TCRs in our dataset, we performed exact CDR3β (or CDR3α) amino acid sequence matching between VDJdb-derived SARS-CoV-2-specific TCR sequences and our experimentally detected TCR repertoires, an approach similar to that used in previous studies (22).

Reinfected individuals exhibited significantly elevated SARS-CoV-2-specific IgG titers compared to primary infection (PI) cases (*p* < 0.001), indicating antibody response potentiation through prior antigen exposure. This aligns with evidence of robust memory B-cell reactivation in convalescence, supporting accelerated humoral recall responses upon re-exposure (28, 29). However, while humoral immunity contributes to viral control, memory B-cell persistence appears limited beyond 5~8 months (15). Crucially, emerging evidence highlights T-cell-mediated immunity as essential for durable protection (15, 29, 30). The adaptive immune system relies on T-cell receptor (TCR) diversity, generated through V(D)J recombination (31), enables recognition of MHC-presented antigens and forms the molecular basis of adaptive immunity (32, 33). Despite its mechanistic importance, direct evidence linking TCR dynamics to SARS-CoV-2 reinfection remains scarce.

We observed reduced TCR diversity alongside increased clonality in both PI and RI groups compared to HC, consistent with antigen-driven clonal expansion (34). TCR diversity and specificity are central to adaptive immunity (35), playing a critical role in viral clearance. Upon antigenic stimulation, T cells with identical TCRs undergo rapid clonal expansion, which promotes the elimination of infected cells but also leads to a reduction in overall TCR diversity (34).

Notably, the lack of significant diversity differences between PI and RI suggests that reinfection does not further diminish TCR diversity, potentially due to memory T-cell responses preventing additional repertoire contraction (36). Intriguingly, TCR repertoire analysis revealed distinct clonal expansion patterns between reinfected and primary infection groups, implying that repeated antigen exposure may drive unique T-cell response dynamics. These findings raise important questions about how TCR remodeling influences clinical outcomes and the durability of immune protection, warranting further investigation to clarify these relationships.

Analysis of TCRα pairings revealed group-specific signatures: TRAV27/TRAJ42 characterized RI, TRAV24/TRAJ42 dominated PI, and TRAV35/TRAJ42 prevailed in HC. Conversely, the TRBV6-4/TRBD2/TRBJ2-3 β-chain motif was conserved across all groups, suggesting a structural role in antigen recognition and possible cross-reactive memory T-cell involvement. Since most circulating T cells express TCRs composed of α and β heterodimers, whose antigen specificity is determined by V(D)J recombination (37), this shared TCRβ motif may reflect cross-reactive memory T cells, potentially contributing to heterologous immunity.

Finally, we novelty applied the Mfuzz clustering method to analyze temporal trends in CDR3 sequences, identifying dynamic patterns associated with disease progression. HC-exclusive clusters exhibited motifs linked to regulatory T cell (Treg) activity, implying a role in memory or immune regulation shaped by post-infection homeostasis. These findings suggest that Treg-associated TCR signatures may contribute to immune protection against SARS-CoV-2 reinfection. Previous studies have shown that SARS-CoV-2 exhibits population-specific immune evasion strategies, underscoring the impact of host genetic background on immune protection (38).

Our study reveals distinct TCR repertoire signatures associated with SARS-CoV-2 reinfection, characterized by reduced clonal diversity, antigen-driven expansion, cohort-specific V(D)J recombination patterns, and exclusive CDR3 AA sequence clusters. The HC-enriched TCR clusters likely represent protective memory T-cell populations, whereas the PI/RI groups exhibited repertoire dynamics consistent with antigen-specific selection pressure. Notably, the significant divergence between RI and HC repertoires suggests compromised T-cell immunity in reinfected individuals, potentially explaining their susceptibility to recurrent infection. We suppose that these TCR sequences represent clonotype subsets that confer SARS-CoV-2 immunity, and that their depletion or dysregulation may contribute to the pathogenesis of reinfection. This interpretation is consistent with other viral models, such as influenza, in which T-cell-mediated immunity has been shown to play a critical role in protection against reinfection (39, 40). These findings advance our mechanistic understanding of recall T-cell responses in reinfection and inform rational vaccine design and immunotherapeutic strategies.

While this study enhances our understanding of T-cell responses in reinfection, several limitations should be noted. First, as a curated database, VDJdb is subject to coverage bias and may overrepresent certain epitopes while underrepresenting others. Second, TCR specificity inferences rely on sequence similarity and previously reported associations in VDJdb and have not been experimentally validated in our cohort. Furthermore, functional assays, such as TCR specificity testing and epitope mapping, are needed to confirm the protective role of the identified T-cell clusters and to elucidate the mechanistic basis of their effects.

## Data Availability

The original contributions presented in the study are publicly available. These data can be accessed through the NCBI repository (Bioproject Accession Number: PRJNA1346690) at the following link: http://www.ncbi.nlm.nih.gov/bioproject/1346690.
