# Supplementary material for: Distinct characteristics of T cell receptor repertoire associated with the SARS-CoV-2 reinfection
Source: Front Immunol. 2025 Oct 23;16:1680089. doi: 10.3389/fimmu.2025.1680089 (PMC12589064; doi:10.3389/fimmu.2025.1680089)
Supplement: Supplementary file 1 [file Table1.docx]

**Distinct characteristics of T cells receptor repertoire associated with the SARS-CoV-2 reinfection**

Liling Zeng^1^; Li Liu^2^; Baolin Ren^1^; Bing Feng^1^; Xudong Lai^2^; Xunxi Lai^2^; Zhimin Chen^3^*; Yihui Huang^4^*; Wenxin Hong^4*^

^1^ State Key Laboratory of Traditional Chinese Medicine Syndrome, The Second Affiliated Hospital of Guangzhou University of Chinese Medicine (Guangdong Provincial Hospital of Chinese Medicine), The Second Clinical Medical College of Guangzhou University of Chinese Medicine, Guangzhou 510120, China;

^2^ Guangzhou Red Cross Hospital, Jinan University, Guangzhou 510000, China;

^3^ Guangdong Polytechnic Normal University, Guangzhou 510665, China;

^4^ Guangzhou Eighth People’s Hospital, Guangzhou Medical University, Guangzhou 510060, China.

* Li Liu and Liling Zeng contributed equally to this work.

*Zhimin Chen, Yihui Huang and Wenxin Hong are the corresponding authors.

Email to Wenxin Hong: [winsonhong@126.com](mailto:winsonhong@126.com). Guangzhou Eighth People’s Hospital, Guangzhou Medical University, Guangzhou 510060, China.

**A. Supplementary methods**

**1. Classification of COVID-19 Severity in Adults Based on WHO Criteria.**

| Mild  disease |  | Symptomatic patients meeting the case definition for COVID-19 without evidence of viral pneumonia or hypoxia. |
| --- | --- | --- |
| Moderate  disease | Pneumonia | with clinical signs of pneumonia (fever, cough, dyspnoea, fast breathing) but no signs of severe pneumonia, including SpO2 ≥ 90% on room air. |
| Severe  disease | Severe pneumonia | with clinical signs of pneumonia (fever, cough, dyspnoea) plus one of the following: respiratory rate > 30 breaths/min, severe respiratory distress, or SpO2 < 90% on room air. |
| Critical  disease | Acute respiratory  distress syndrome  (ARDS) | PaO2/FiO2 ≤ 300 mmHg with PEEP or CPAP ≥ 5 cmH2O or need for invasive ventilation. |

1. **Schematic figure of the bioinformatic workflow**


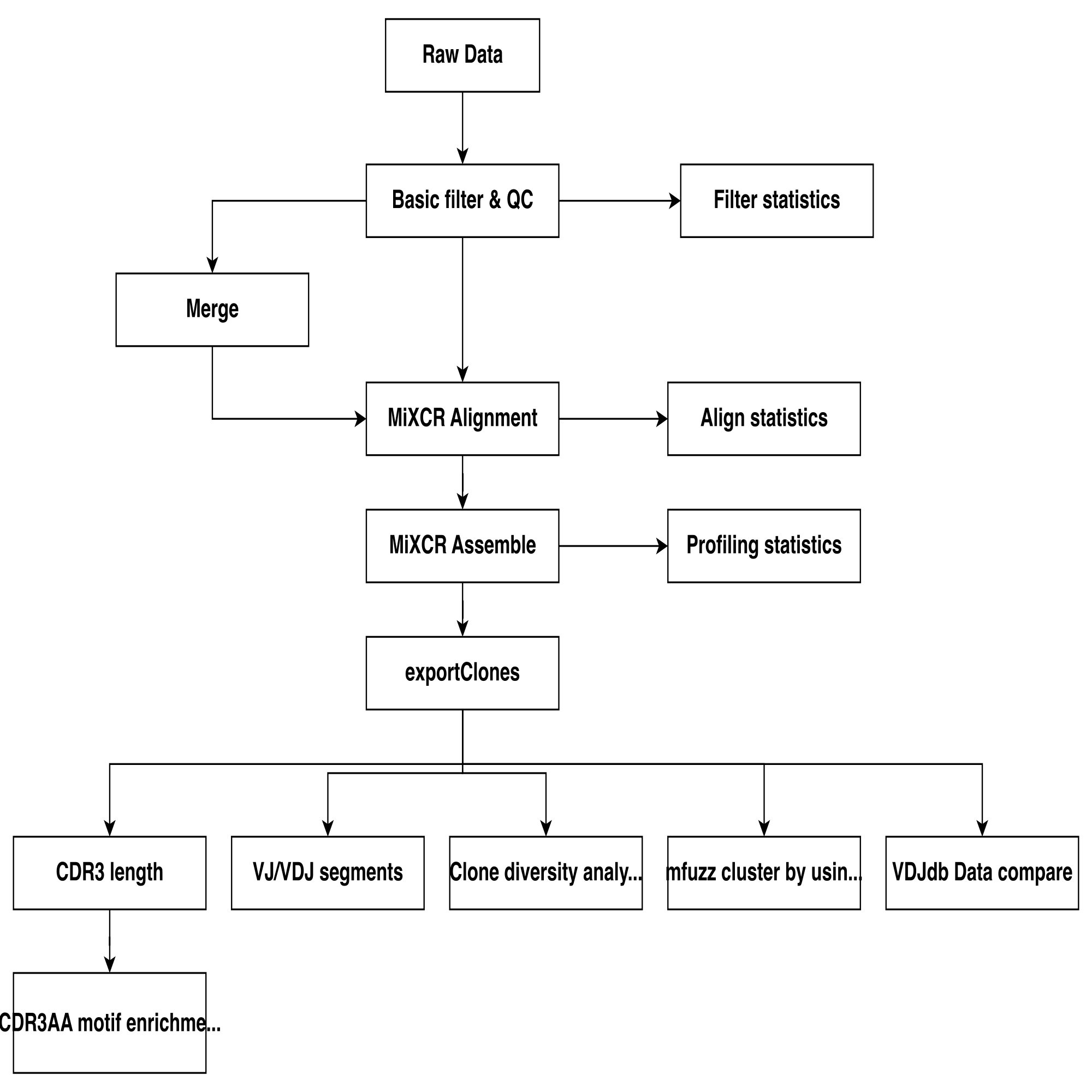


1. **Description of Diversity indices**

Chao1 and ChaoE: The Chao1 index estimates species richness by predicting the total number of unique clonotypes in a sample, including undetected rare species. In contrast, ChaoE (Chao entropy) approximates the Shannon diversity index and is more robust in undersampled communities—such as those often encountered in immune repertoire studies. These indices are complementary: Chao1 reflects richness, while ChaoE assesses entropy.

Calculation methods: Clonal frequencies for α and β chains were compiled into a sample-by-clonotype matrix. Using the R package vegan (v2.7-1), the richness_estimates() function computed multiple TCR diversity indices. Chao1 was directly extracted from these results, and the coverage() function was used to derive ChaoE based on Chao1 values.

Shannon and Inverse Simpson indices, which integrate both richness and evenness, were also calculated using richness_estimates() from vegan.

DE50 represents the percentage of dominant clonotypes needed to comprise 50% of the total T-cell population, with lower values indicating higher clonality. Using base R, clones were ranked by frequency, and the cumulative sum was computed with cumsum(). The point where cumulative abundance reached 50% was identified, and DE50 was calculated as this index divided by the total number of clones. A high DE50 suggests uniform clonal abundance, while a low value indicates oligoclonality.

Gini Coefficient: This metric quantifies inequality in clonal abundance (0 = perfectly even, 1 = perfectly uneven). It was computed directly using the R package ineq (v0.2-13) from the clonotype matrix with default parameters.

1. **Repertoire size normalization method**

In our study, to address the variation in sequencing depth and repertoire size across samples, we applied a consistent normalization procedure to all TCR repertoire data before conducting any diversity or clonality analyses (including calculations of Chao1, ChaoE, Shannon, clonality indices, and so on). Our method involves converting the raw read count of each TCR clone into a proportional frequency (relative abundance) within each sample. Specifically, for each sample, we summed the absolute counts of all TCR clones to obtain a total repertoire count. Each clone’s abundance was then divided by this total to obtain its normalized frequency.

1. **V(D)J Allele Usage Analysis**

Allele usage was analyzed based on normalized frequency, not raw read counts, to account for differences in sequencing depth and repertoire size across samples. Only reads with unambiguously assigned V and J genes were included. Ambiguous or unassignable alleles were filtered out prior to analysis. Usage frequency for each allele was calculated as the proportion of reads containing that allele relative to the total number of productively rearranged and assigned T cell receptor sequences per sample.

1. **CDR3 Analysis**

CDR3 sequences were inferred from productive TCR rearrangements using the MiXCR pipeline. Only in-frame sequences without stop codons were retained. CDR3 amino acid sequences were then used for subsequent analyses. the details as follows:


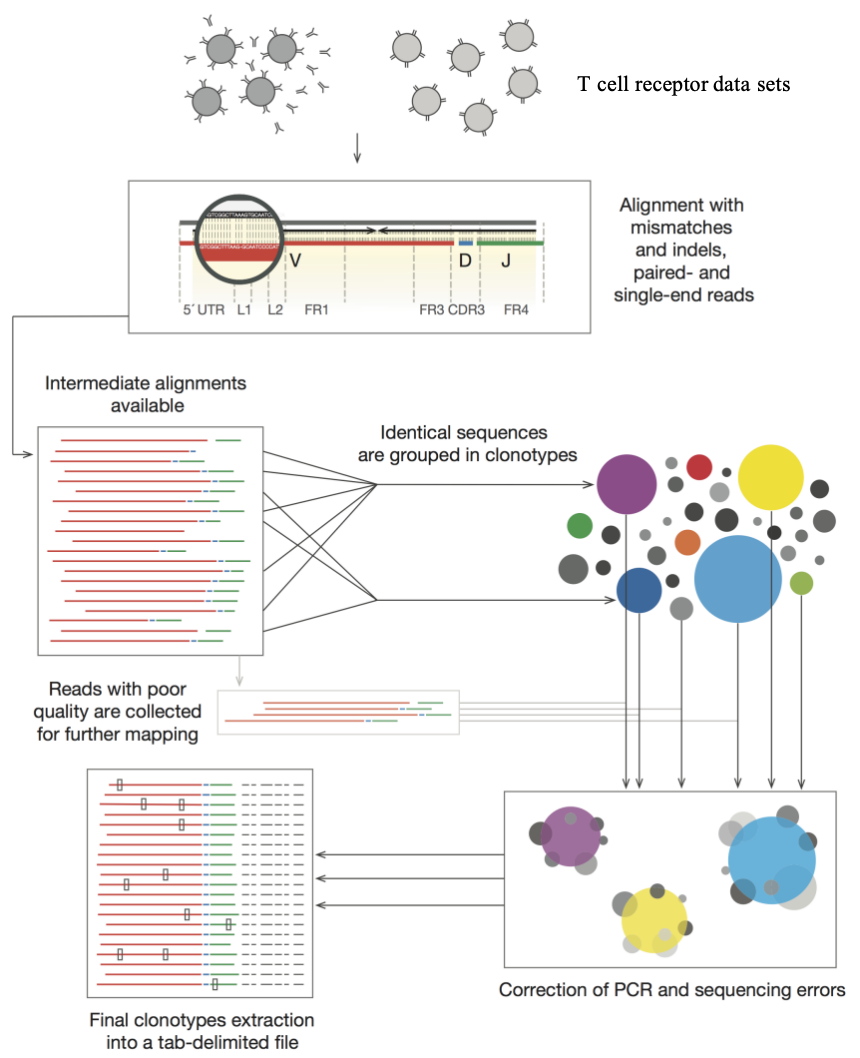


1. **Principal Component Analysis (PCA)**

Principal component analysis (PCA) was performed to reduce the dimensionality and visualize the variability among samples based on TCR repertoire diversity. The input variables consisted of multiple diversity indices, such as richness, evenness, divergence, and clonality, calculated from the normalized frequency information of both α and β chains for each sample. These indices collectively captured distinct aspects of TCR diversity and were used as features in the PCA.

Using the prcomp function in R (v3.6.2), the diversity index matrix was centered by subtracting the mean of each variable. Principal components were then derived from the covariance matrix to identify major axes of variation across samples. The first two principal components (PC1 and PC2), which captured the largest proportions of variance, were used to project the high-dimensional diversity profiles into a two-dimensional space. This allowed for the visualization of similarities and differences between samples or experimental groups based on their overall diversity structure.

The resulting PCA plot provided an intuitive summary of diversity patterns, while additional visualizations such as box plots and violin plots along each principal component further illustrated group-specific distributions and helped interpret biological variation in TCR repertoire characteristics.

1. **Mfuzz clustering and "temporal trends"**

We applied the Mfuzz clustering method, based on the Fuzzy C-Means (FCM) algorithm, to identify gradual and overlapping temporal patterns in noisy TCR repertoire time-series data. Clustering was performed using the clusterData() function in R on normalized clonal frequency data. The number of clusters was set to 10 to balance pattern resolution against over-fragmentation, based on cluster stability assessment and biological relevance. The soft clustering method "mfuzz" was used, with the fuzzifier parameter automatically set according to package guidelines to ensure appropriate cluster cohesion and separation.

We hypothesized that certain TCR clones show systematic frequency changes across post-infection timepoints: initial infection (PI), convalescence (HC), and reinfection (RI). To group clones with similar dynamics, each clone’s frequency trajectory was z-score normalized to emphasize shape over magnitude. Clones were assigned to clusters based on membership values, reflecting their degree of fit to each trend pattern.

Mfuzz allows soft clustering, where a clone may belong to multiple clusters, a flexibility suited to capturing biological continuity in immune responses, as commonly applied in gene expression time-series studies.

1. **Supplementary Results**

**Table 1. Demographic and Clinicopathologic Features of the Study Participants**

|  | PI  (n=18) | RI  (n=18) | Healthy  (n = 12) | *p*-value |
| --- | --- | --- | --- | --- |
| Age, years | 31.44±11.77 | 26.78±5.24 | 27.33±9.09 | 0.134 |
| Sex |  |  |  |  |
| Male | 9 | 9 | 6 | 1.000 |
| White blood cells (10^9^/L) | 6.93±2.77 | 7.00±1.44 |  | 0.927 |
| Number of neutrophils (10^9^/L) | 5.24±2.60 | 5.20±1.49 |  | 0.945 |
| Number of lymphocytes (10^9^/L) | 0.92±0.44 | 1.06±0.58 |  | 0.423 |
| platelet(10^9^/L) | 206.50±35.08 | 221.61±50.70 |  | 0.306 |
| Red blood cells(10^12^/L) | 4.79±0.51 | 5.01±0.58 |  | 0.244 |
| Hemoglobin (g/L) | 195.67±230.52 | 143.89±13.19 |  | 0.348 |
| Course of disease | 2.11±0.83 | 1.83±0.38 |  | 0.207 |
| Clinical symptoms |  |  |  |  |
| fever | 18（100%） | 18（100%） |  | 1.000 |
| Sore throat | 17（94.4%） | 18（100%） |  | 1.000 |
| cough | 12（66.7%） | 15（83.3%） |  | 0.443 |
| Expectoration | 11（61.6%） | 10（55.6%） |  | 1.000 |
| Nasal congestion | 1（5.6%） | 0（100%） |  | 1.000 |
| Runny nose | 1（5.6%） | 1（5.6%） |  | 1.000 |
| Headache | 15（83.3%） | 11（61.1%） |  | 0.264 |
| muscle ache | 14（77.8%） | 11（61.1%） |  | 0.471 |
| Joint pain | 14（77.8%） | 11（61.1%） |  | 0.471 |
| Loss of appetite | 8（44.4%） | 12（66.7%） |  | 0.315 |
| Vomit | 0（100%） | 1（5.6%） |  | 1.000 |
| Chest tightness | 0（100%） | 0（100%） |  | 1.000 |
| Shortness of breath | 0（100%） | 0（100%） |  | 1.000 |
| diarrhea | 0（100%） | 1（5.6%） |  | 1.000 |
| Severity | Moderate | Moderate |  |  |
| Complication | N | N |  |  |

HC, Healthy convalescents; PI, primary SARS-CoV-2 infection; RI, reinfected SARS-CoV-2.

**Table 2.Top unique V(D)J pairs per cohort**

| **Unique TRA pairs** | | | **Unique TRB pairs** | | |
| --- | --- | --- | --- | --- | --- |
| **PI** | **HC** | **RI** | **PI** | **HC** | **RI** |
| TRAV12-1_TRAJ28 | TRAV1-1_TRAJ42 | TRAV13-1_TRAJ13 | TRBV11-2_TRBD2_TRBJ1-1 | TRBV27_TRBD2_TRBJ2-7 | TRBV11-1_TRBD1_TRBJ2-1 |
| TRAV12-2_TRAJ49 | TRAV1-2_TRAJ30 | TRAV13-1_TRAJ42 | TRBV27_TRBD1_TRBJ2-1 | TRBV28_TRBD1_TRBJ2-7 | TRBV12-3_TRBD1_TRBJ2-1 |
| TRAV21_TRAJ10 | TRAV20_TRAJ42 | TRAV17_TRAJ20 | TRBV6-1_TRBD2_TRBJ2-3 | TRBV4-1_TRBD1_TRBJ2-7 | TRBV12-3_TRBD1_TRBJ2-2 |
| TRAV24_TRAJ42 | TRAV29DV5_TRAJ42 | TRAV2_TRAJ30 | TRBV6-5_TRBD1_TRBJ1-5 | TRBV6-1_TRBD1_TRBJ2-7 | TRBV12-4_TRBD1_TRBJ2-2 |
| TRAV9-2_TRAJ42 | TRAV3_TRAJ30 | TRAV21_TRAJ34 | TRBV7-2_TRBD1_TRBJ2-1 | TRBV6-4_TRBD1_TRBJ2-3 | TRBV27_TRBD1_TRBJ1-1 |
|  | TRAV41_TRAJ42 | TRAV22_TRAJ30 | TRBV7-3_TRBD1_TRBJ1-1 |  | TRBV5-1_TRBD1_TRBJ2-1 |
|  | TRAV41_TRAJ49 | TRAV5_TRAJ10 |  |  | TRBV5-1_TRBD2_TRBJ2-2 |
|  |  | TRAV5_TRAJ29 |  |  | TRBV6-1_TRBD1_TRBJ1-1 |
|  |  |  |  |  | TRBV6-5_TRBD1_TRBJ2-2 |
|  |  |  |  |  | TRBV9_TRBD1_TRBJ2-3 |

**Table 3. Overlap of TCRα VJ Alleles and Clonotypes of Interest**

| **TRAV1-1/TRAJ42** | **TRAV1-2/TRAJ33** | **TRAV12-2/TRAJ49** | **TRAV21/TRAJ49** | **TRAV24/TRAJ42** | **TRAV27/TRAJ42** | **TRAV35/TRAJ42** | **TRAV5/TRAJ10** |  |
| --- | --- | --- | --- | --- | --- | --- | --- | --- |
| CAVYGGSQGNLIF | CAVRDSNYQLIW | CAVNTGNQFYF | CAVNTGNQFYF | CAVYGGSQGNLIF | CAVYGGSQGNLIF | CAVYGGSQGNLIF | CAESFTGGGNKLTF |  |
| CAGGSQGNLIF | CAVMDSNYQLIW | CAVTGNQFYF | CAVTGNQFYF | CAGGSQGNLIF | CAGGSQGNLIF | CAGGSQGNLIF | CAASTGGGNKLTF |  |
| CAAGGSQGNLIF | CAAMDSNYQLIW | CAVITNTGNQFYF | CAVITNTGNQFYF | CAAGGSQGNLIF | CAAGGSQGNLIF | CAAGGSQGNLIF | CAGVTGGGNKLTF |  |
| CAVLNYGGSQGNLIF | CAVTDSNYQLIW | CAVSNTGNQFYF | CAVSNTGNQFYF | CALGGSQGNLIF | CALGGSQGNLIF | CAGLNYGGSQGNLIF | CAVAGGGNKLTF |  |
| CALGGSQGNLIF | CAVLDSNYQLIW | CAATGNQFYF | CAATGNQFYF | CAPGGSQGNLIF | CAPGGSQGNLIF | CAVLNYGGSQGNLIF | CAGGGNKLTF |  |
| CAPGGSQGNLIF | CAALDSNYQLIW | CAASNTGNQFYF | CAASNTGNQFYF | CAAMNYGGSQGNLIF | CAAMNYGGSQGNLIF | CAVGGSQGNLIF | CVVTGGGNKLTF |  |
| CAAMNYGGSQGNLIF | CAVSDSNYQLIW | CAALGTGNQFYF | CAALGTGNQFYF | CAWGGSQGNLIF | CAGLNYGGSQGNLIF | CAVMNYGGSQGNLIF | CAPTGGGNKLTF |  |
| CAVGGSQGNLIF | CAPLDSNYQLIW | CAVRGTGNQFYF | CAVRGTGNQFYF | CASGGSQGNLIF | CAVLNYGGSQGNLIF | CAALNYGGSQGNLIF | CAAPTGGGNKLTF |  |
| CAVMNYGGSQGNLIF | CAVREGSNYQLIW | CALTNTGNQFYF | CALTNTGNQFYF | CAGLNYGGSQGNLIF | CAVGGSQGNLIF | CAAANYGGSQGNLIF | CAYTGGGNKLTF |  |
| CAALNYGGSQGNLIF | CAVYDSNYQLIW | CAVDGQFYF | CAVDGQFYF | CAAKNYGGSQGNLIF | CAVMNYGGSQGNLIF | CAGPNYGGSQGNLIF |  |  |
| CAAANYGGSQGNLIF | CAGRDSNYQLIW | CAVYTGNQFYF | CAVYTGNQFYF | CAFMNYGGSQGNLIF | CAALNYGGSQGNLIF | CAGRMNYGGSQGNLIF |  |  |
| CAGPNYGGSQGNLIF | CARMDSNYQLIW | CALNTGNQFYF | CALNTGNQFYF | CASPNYGGSQGNLIF | CAAANYGGSQGNLIF | CAGSNYGGSQGNLIF |  |  |
| CAVRGGGSQGNLIF | CAVRRGNYQLIW | CAANTGNQFYF | CAANTGNQFYF | CALLNYGGSQGNLIF | CAGPNYGGSQGNLIF | CAGANYGGSQGNLIF |  |  |
| CAWGGSQGNLIF | CASVDSNYQLIW | CAVRRNTGNQFYF | CAVRRNTGNQFYF | CASMNYGGSQGNLIF | CAVRGGGSQGNLIF | CAGRNYGGSQGNLIF |  |  |
| CAVSGGSQGNLIF | CAVTNSNYQLIW | CAALNTGNQFYF | CAALNTGNQFYF | CASQNYGGSQGNLIF | CAGRMNYGGSQGNLIF | CAGFNYGGSQGNLIF |  |  |
| CASGGSQGNLIF | CAVNMDSNYQLIW | CAVASNTGNQFYF | CAVASNTGNQFYF | CAFYYGGSQGNLIF | CAGSNYGGSQGNLIF | CAGMNYGGSQGNLIF |  |  |
| CAVSNYGGSQGNLIF | CAVGSNYQLIW | CAPSNTGNQFYF | CAPSNTGNQFYF | CAPMNYGGSQGNLIF | CAGANYGGSQGNLIF | CAGTNYGGSQGNLIF |  |  |
| CAARNYGGSQGNLIF | CAGTDSNYQLIW | CATTGNQFYF | CATTGNQFYF | CASLNYGGSQGNLIF | CAGRNYGGSQGNLIF | CAAPGGSQGNLIF |  |  |
| CAVRDRGSQGNLIF | CAVRDLDSNYQLIW | CAVSPTGNQFYF | CAVSPTGNQFYF | CAYSYGGSQGNLIF | CAGFNYGGSQGNLIF | CAGAYYGGSQGNLIF |  |  |
| CAVRGGSQGNLIF | CAVRSNYQLIW | CADPNTGNQFYF | CADPNTGNQFYF | CALENYGGSQGNLIF | CAGMNYGGSQGNLIF | CAGQNYGGSQGNLIF |  |  |
| CAVKDYGGSQGNLIF | CAMRDSNYQLIW | CATNTGNQFYF | CATNTGNQFYF | CALEGSQGNLIF | CAGTNYGGSQGNLIF | CAGKNYGGSQGNLIF |  |  |
| CAVRAYGGSQGNLIF | CAVRPLMDSNYQLIW | CAGRTGNQFYF | CAGRTGNQFYF | CACLNYGGSQGNLIF | CAAPGGSQGNLIF | CAGHNYGGSQGNLIF |  |  |
|  | CALVDSNYQLIW | CAVGAGNQFYF | CAVGAGNQFYF | CAINYGGSQGNLIF | CAGAYYGGSQGNLIF | CAGLLYGGSQGNLIF |  |  |
|  | CAGDSNYQLIW | CAINTGNQFYF | CAINTGNQFYF | CAPSYGGSQGNLIF | CAGQNYGGSQGNLIF | CAAKNYGGSQGNLIF |  |  |
|  | CVNMDSNYQLIW | CAGPTGNQFYF | CAGPTGNQFYF | CAPENYGGSQGNLIF | CAGKNYGGSQGNLIF | CAVSGGSQGNLIF |  |  |
|  | CAVRVDSNYQLIW | CADSNTGNQFYF | CADSNTGNQFYF | CAIPNYGGSQGNLIF | CAGHNYGGSQGNLIF | CAVSNYGGSQGNLIF |  |  |
|  |  | CASNTGNQFYF | CASNTGNQFYF | CALAGSQGNLIF | CAGLLYGGSQGNLIF | CAARNYGGSQGNLIF |  |  |
|  |  | CAVPLTGNQFYF | CAVPLTGNQFYF |  | CAGARGGSQGNLIF | CAAQNYGGSQGNLIF |  |  |
|  |  | CALDTGNQFYF | CALDTGNQFYF |  | CAGEGSQGNLIF | CAGQLYGGSQGNLIF |  |  |
|  |  | CAGETGNQFYF | CAVRPSGNQFYF |  | CAGTDYGGSQGNLIF | CAASSNYGGSQGNLIF |  |  |
|  |  | CALRTGNQFYF | CAVGADTGNQFYF |  | CAVQNYGGSQGNLIF | CAGFLNYGGSQGNLIF |  |  |
|  |  | CAVR | CAVRPWGNQFYF |  |  | CAGQLFGGSQGNLIF |  |  |
|  |  | CVVNRDTGNQFYF | CAALGNQFYF |  |  |  |  |  |
|  |  |  | CALGSGNQFYF |  |  |  |  |  |
|  |  |  | CAISTGNQFYF |  |  |  |  |  |
|  |  |  | CALGGTGNQFYF |  |  |  |  |  |
|  |  |  | CAVADGNQFYF |  |  |  |  |  |
|  |  |  | CAADYTGNQFYF |  |  |  |  |  |
|  |  |  | CGANTGNQFYF |  |  |  |  |  |
|  |  |  | CAVLVPGNQFYF |  |  |  |  |  |

**Table 4. Overlap of TCRβ V(D)J Alleles and Clonotypes of Interest**

| **TRBV11-1/TRBD1/TRBJ2-1** | | **TRBV27/TRBD2/TRBJ2-7** | **TRBV5-1/TRBD1/TRBJ2-7** | **TRBV6-4/TRBD2/TRBJ2-3** | | **TRBV7-2/TRBD1/TRBJ2-1** | **TRBV7-6/TRBD1/TRBJ2-3** | | | | | |  |
| --- | --- | --- | --- | --- | --- | --- | --- | --- | --- | --- | --- | --- | --- |
| CASSLTGYNEQFF | | CASSGGYEQYF | CASSGGYEQYF | CASSLRGTDTQYF | CASSLGQSYEQYF | CASSLTGYNEQFF | CASSLRGTDTQYF | | | | | |  |
| CASSLGQSSYNEQFF | | CASSLAGGSYEQYF | CASSLAGGSYEQYF | CASSPTGGTDTQYF | CASSLQGGTYEQYF | CASSLGQSSYNEQFF | CASSPTGGTDTQYF | | | | | |  |
|  | | CASSLASGYEQYF | CASSLASGYEQYF | CASSGTSGSTDTQYF | CASSLVQGSYEQYF | CASSLGGDEQFF | CASSLGGSSTDTQYF | | | | | |  |
|  | | CASSLVTGEQYF | CASSLVTGEQYF | CASSVAGGTDTQYF | CASSEGGGYEQYF | CASSVDRGAYNEQFF | CASSPGTDTQYF | | | | | |  |
|  | | CASSPGTSYEQYF | CASSPGTSYEQYF | CASSPTSGSTDTQYF | CASSPGTTYEQYF | CASSLAGDDYNEQFF | CASSLAGGLTDTQYF | | | | | |  |
|  | | CASSLAGGYEQYF | CASSLAGGYEQYF | CASSGSGGATDTQYF | CASSSQGGEQYF | CASSFTGSSYNEQFF | CASSPGDTQYF | | | | | |  |
|  | | CASSLSGSYEQYF | CASSLSGSYEQYF | CASSLAGESDTQYF | CASSSGTGVDYEQYF | CASGGYNEQFF | CASSLGPADTQYF | | | | | |  |
|  | | CASSLGGEQYF | CASSLGGEQYF | CASSERGGPDTQYF | CASSLQGVSYEQYF | CASSSSGYNEQFF | CASSLRQGADTQYF | | | | | |  |
|  | | CASSSGGYEQYF | CASSSGGYEQYF | CASSPGLTDTQYF | CASSLLGGYEQYF | CASSLDRGVYNEQFF | CASSARGDTQYF | | | | | |  |
|  | | CASSLEGHEQYF | CASSLEGHEQYF |  | CASSLGNYEQYF | CASSVSGGAYNEQFF | CASSLGTASTDTQYF | | | | | |  |
|  | | CASSPGTGRYEQYF | CASSPGTGRYEQYF |  | CASSLAQGYEQYF | CASSLSSYNEQFF | CASSLGTGDTDTQYF | | | | | |  |
|  | | CASSLGGGAYEQYF | CASSLGGGAYEQYF |  | CASSLGAGTYEQYF | CASSLGGAGYNEQFF | CASSLGGGADTQYF | | | | | |  |
|  | | CASSLLGGSYEQYF | CASSLLGGSYEQYF |  | CASSPDSEQYF | CASSPPGSYNEQFF | CASSPRGPTDTQYF | | | | | |  |
|  | | CASSLGGEGYEQYF | CASSLGGEGYEQYF |  | CASSLNRGYEQYF | CASSGTGGNEQFF | CASSSRAASTDTQYF | | | | | |  |
|  | | CASSTGSYEQYF | CASSTGSYEQYF |  | CASSVGQGYEQYF | CASSRDYNEQFF | CASSLGGGSTDTQYF | | | | | |  |
|  | | CASSLAGAYEQYF | CASSLAGAYEQYF |  | CASSQQGANEQYF | CASSLGGHGNEQFF | CASSLRGDTQYF | | | | | |  |
|  | | CASSSRTGPYEQYF | CASSSRTGPYEQYF |  | CASSLDSYEQYF | CASSAPSYNEQFF | CASSLGPDTQYF | | | | | |  |
|  | | CASSLTGGGYEQYF | CASSLTGGGYEQYF |  | CASSQGGNEQYF | CASSLGPGQGYNEQFF |  | | | | | |  |
|  | | CASSLGTGYEQYF | CASSLGTGYEQYF |  | CASSLLSGSSYEQYF | CASSLYGATYNEQFF |  | | | | | |  |
|  | | CASSWTSGSYEQYF | CASSLGQAYEQYF |  | CASSLSPREQYF | CASSLDRVSYNEQFF |  | | | | | |  |
|  | | CASSLAGYEQYF | CASSRDRGYEQYF |  | CASSPTGDREQYF | CASSLVGDSYNEQFF |  | | | | | |  |
|  | | CASSLGTSGYEQYF | CASSSGQGYEQYF |  | CASSSSGTATYEQYF | CASSLAGANEQFF |  | | | | | |  |
|  | | CASSPSGPYEQYF | CASSLETGYEQYF |  | CASSLDSPYEQYF | CASSLGPYNEQFF |  | | | | | |  |
|  | | CASSPTSGSYEQYF | CASSPGQGYEQYF |  | CASSFGQGGYEQYF | CASSRTGSSYNEQFF |  | | | | | |  |
|  | | CASSLTSGYEQYF | CASSSGQGVYEQYF |  | CASSSRDRGTYEQYF | CASSPRGNNEQFF |  | | | | | |  |
|  | | CASSFTSGSYEQYF | CASSLAGVEQYF |  | CASSSRQGPYEQYF | CASSLGASSYNEQFF |  | | | | | |  |
|  | | CASSRAGGTYEQYF | CASSLSDSYEQYF |  | CASSPLGTYEQYF | CASSLALAGNEQFF |  | | | | | |  |
|  | | CASSPSSYEQYF | CASSLEQGSYEQYF |  | CASSGQGNYEQYF | CASSLEAGDYNEQFF |  | | | | | |  |
|  | | CASSPLSYEQYF | CASSFGQYEQYF |  | CASRRDRAYEQYF | CASSRDRGEYNEQFF |  | | | | | |  |
|  | | CASSWGGAYEQYF | CASSLGQGPYEQYF |  | CASRGEGSYEQYF | CASSPSGRGGNEQFF |  | | | | | |  |
|  | | CASSLREGLGEQYF | CASSSGTVPYEQYF |  | CASSYTGTEQYF | CASSLSVSSYNEQFF |  | | | | | |  |
|  | | CASSLRSNSYEQYF | CASRSGTGTYEQYF |  | CASSLPGTGDYEQYF | CASSLDSRGNEQFF |  | | | | | |  |
|  | | CASSLGSEQYF | CASSLEGAGYEQYF |  | CASSLGRGAYEQYF | CASSPGSSYNEQFF |  | | | | | |  |
|  | | CASSLRTDSYEQYF | CASSLGYEQYF |  | CASSAGDEQYF | CASSLAQGTYNEQFF |  | | | | | |  |
|  | | CASSPRGGGIYEQYF | CASSPRDRGYEQYF |  | CASSPRTATYEQYF | CASSPGASYNEQFF |  | | | | | |  |
|  | | CASSHGGYEQYF | CASSRDGSYEQYF |  | CASSEGQGYEQYF | CASSPSGVYNEQFF |  | | | | | |  |
|  | | CASKREGSSYEQYF | CASSVRDRTYEQYF |  | CASSETGGYEQYF | CASSSGTGAWNEQFF |  | | | | | |  |
|  | |  |  |  | CASSPMGHEQYF | CASSPRQGKDNEQFF |  | | | | | |  |
|  | |  |  |  | CASSFRTDSYEQYF | CASSPVSYNEQFF |  | | | | | |  |
|  | |  |  |  | CASSSTGDAYEQYF | CASSNRGGNEQFF |  | | | | | |  |
|  | |  |  |  | CASSEATGYEQYF | CASSPGSAVYNEQFF |  | | | | | |  |
|  | |  |  |  |  | CASSFSGGSYNEQFF |  | | | | | |  |
|  | |  |  |  |  | CASSRTGGSYNEQFF |  | | | | | |  |
|  | |  |  |  |  | CASSLAGASSYNEQFF |  | | | | | |  |
|  | | | | | |  |  |  |  |  |  |  |  |


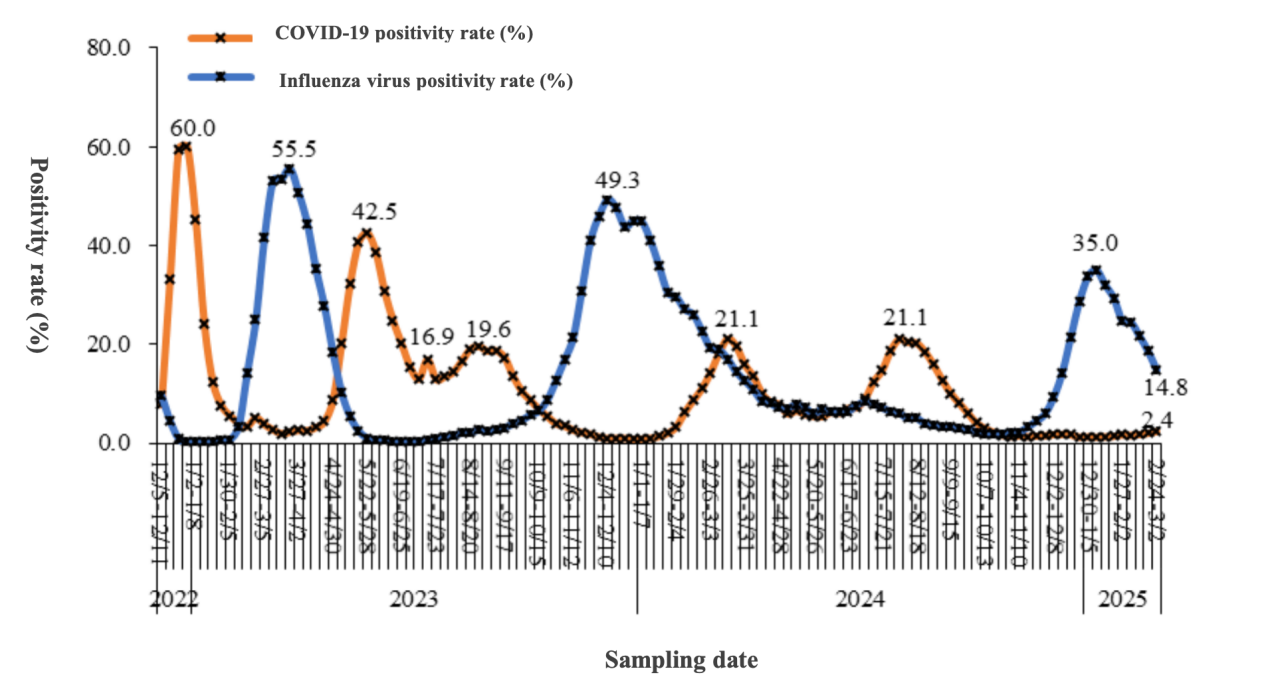


**Figure 1** The changing trend of the positive rates of COVID-19 and influenza viruses in influenza-like cases in sentinel hospitals across China.

Data from <https://www.chinacdc.cn/jksj/xgbdyq/202503/t20250324_305223.html>.


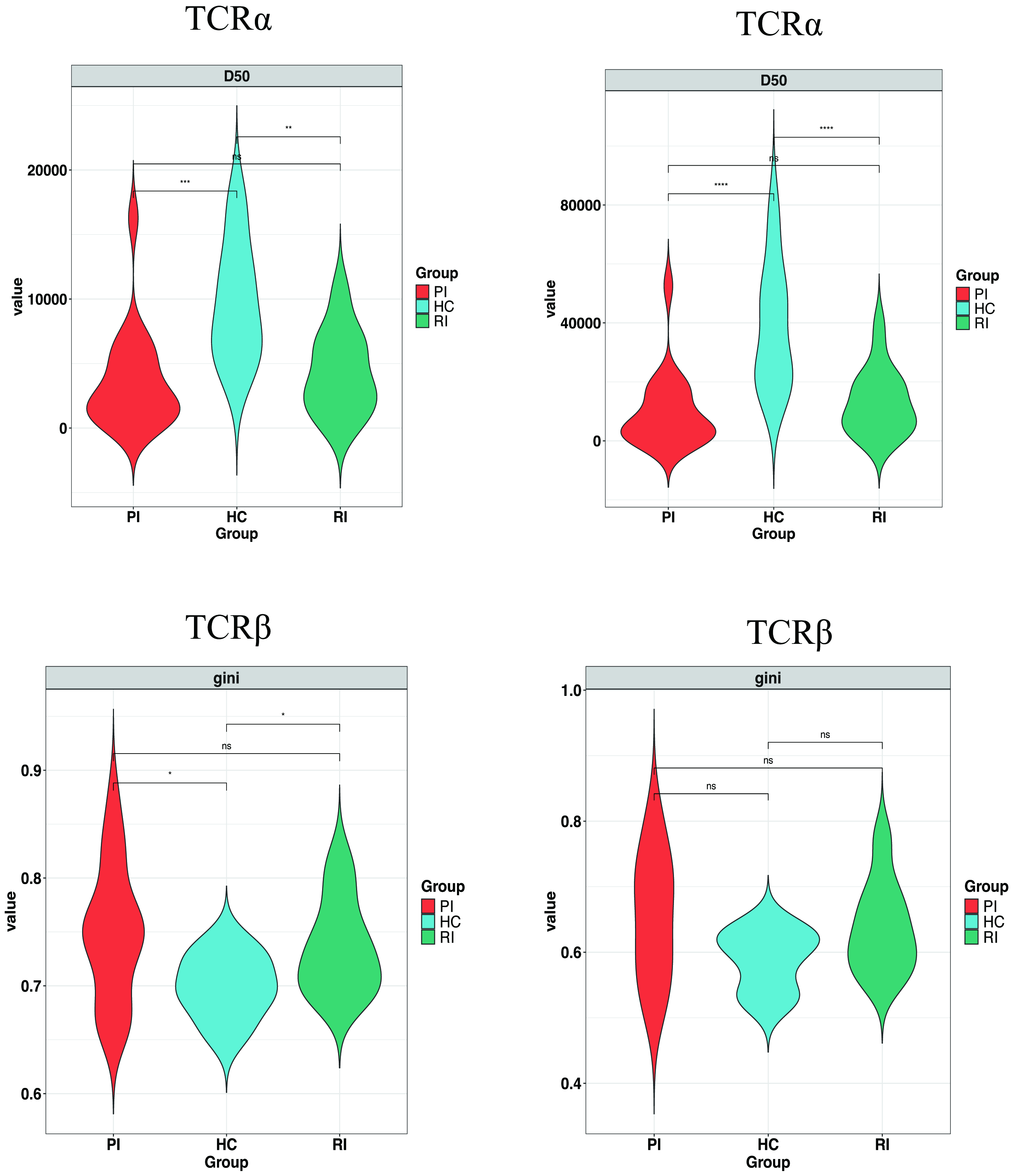


**Figure 2** DE50 and Gini indices for diversity.


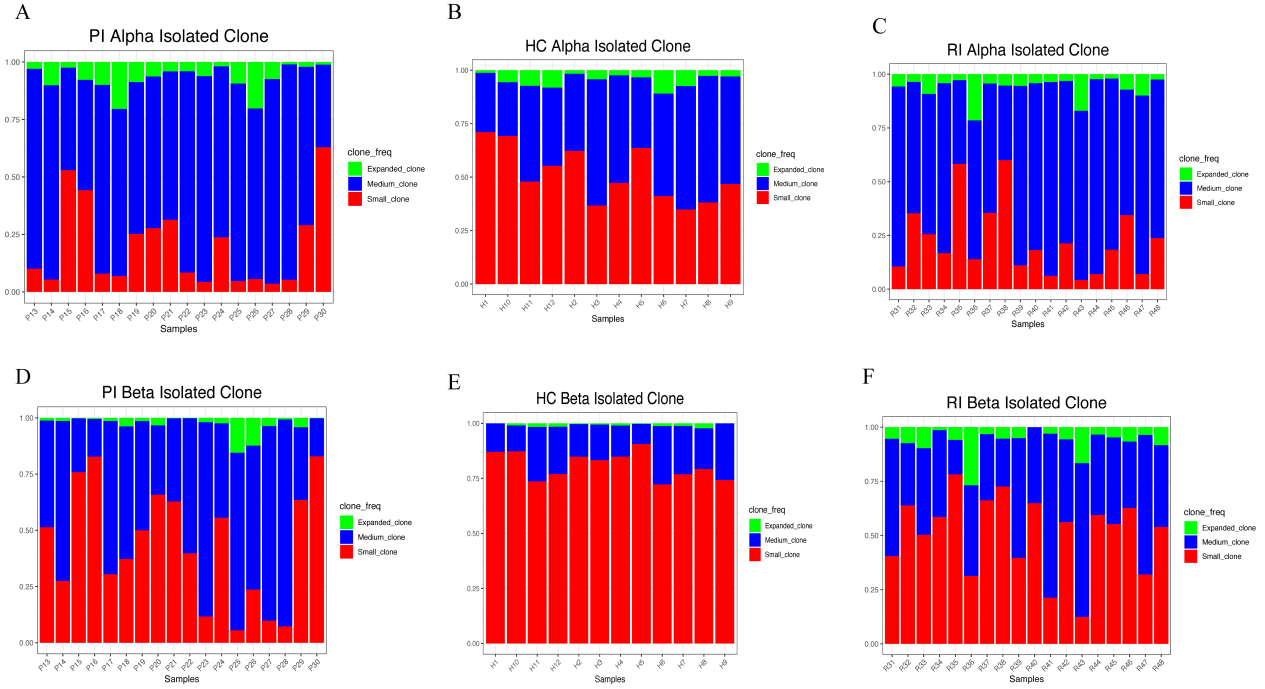


**Figure 3** Distribution frequencies of TCR clonotypes associated with COVID-19 across study groups.


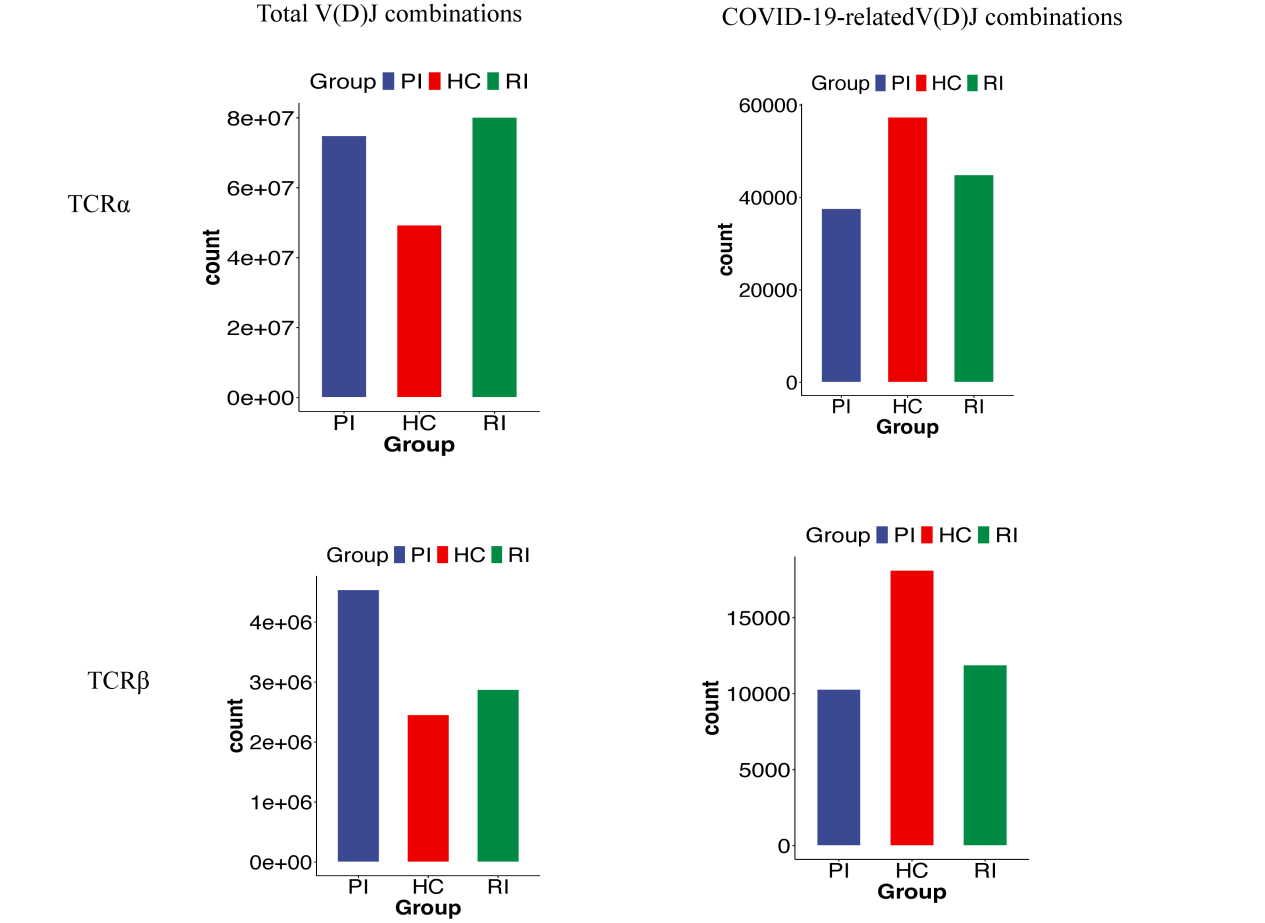


**Figure 4** Distribution of V(D)-J pairings of the TCRαβ chains the in our experimentally detected TCR repertoires and COVID-19-relatived VDJ patterns.


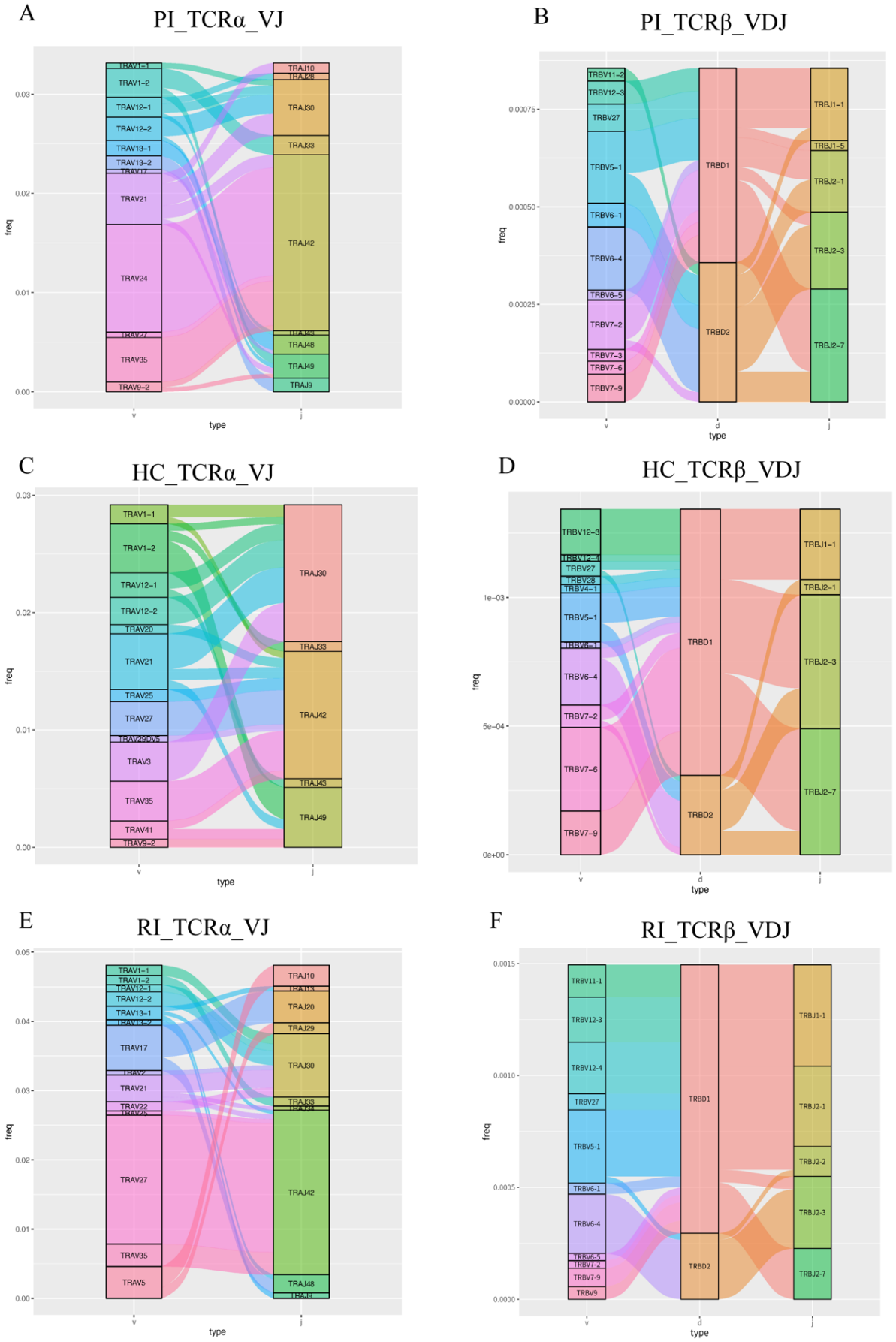


**Figure 5** Overall usage of V, D, and J gene segments in TCRαβ in our experimentally detected TCR repertoires.


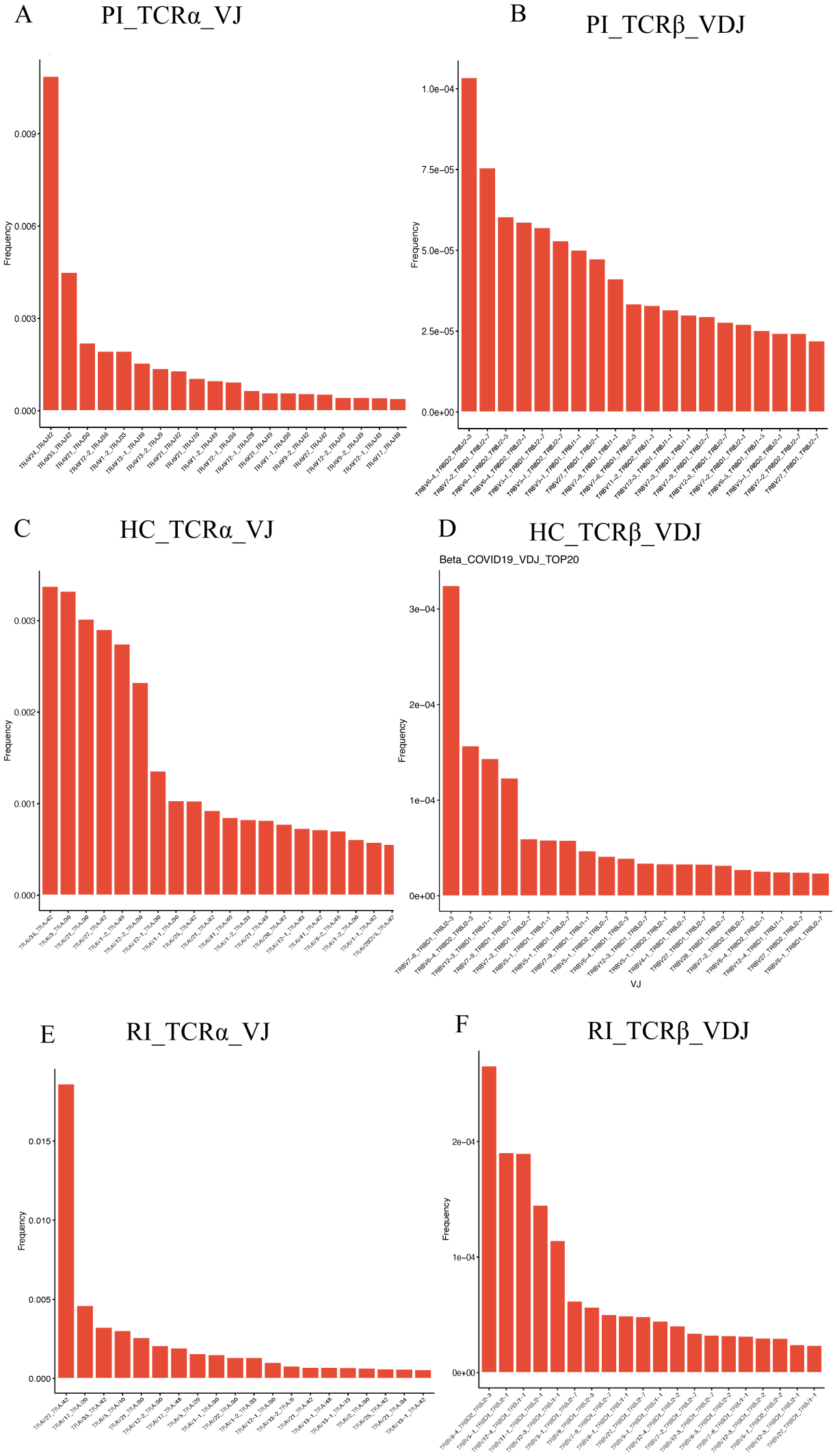


**Figure 6** Histogram of the top 20 COVID-19-associated VDJ patterns for TCRαβ


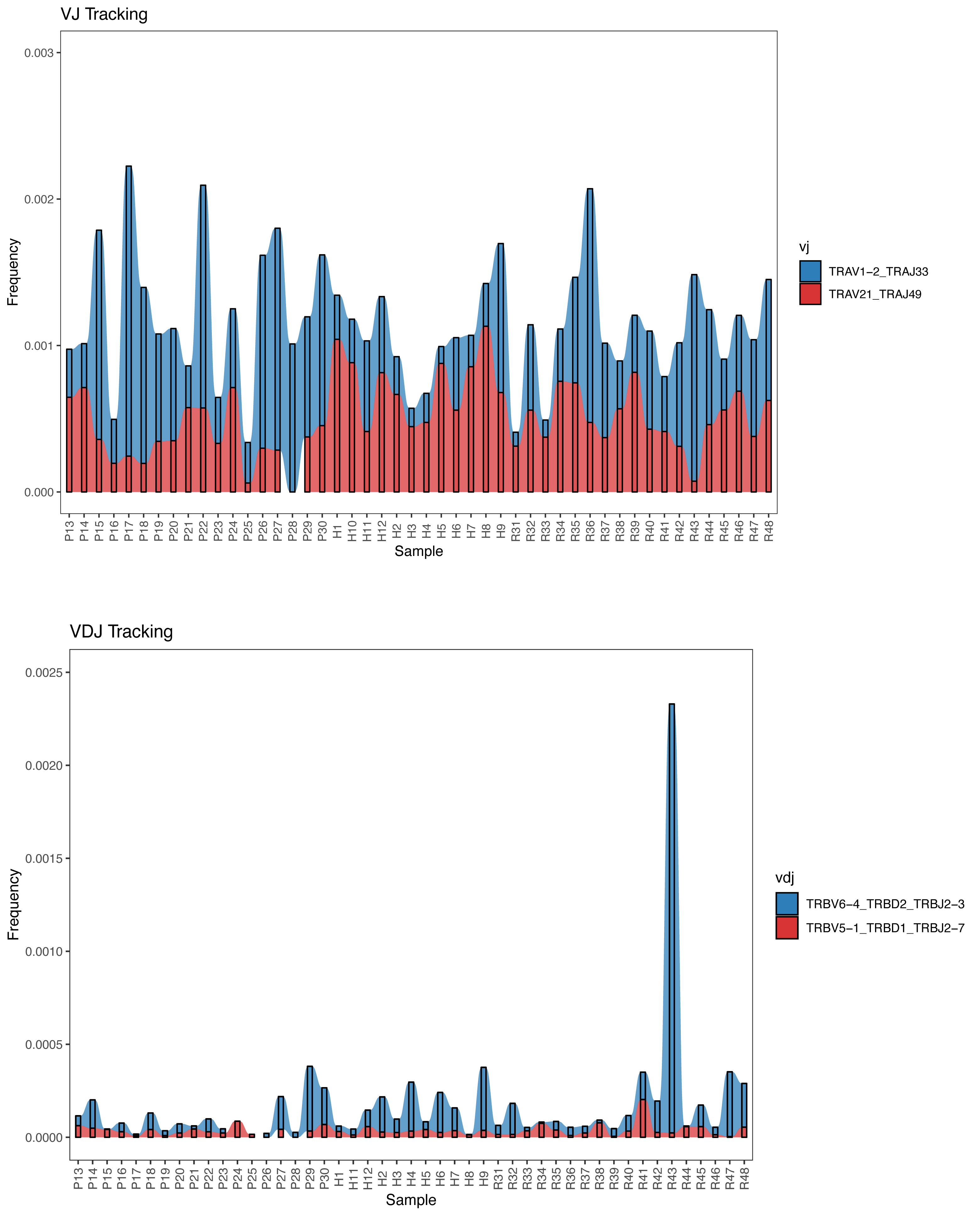


**Figure 7** Persistence of Common TRA/TRB Pairs from Primary Infection through Convalescence to Reinfection


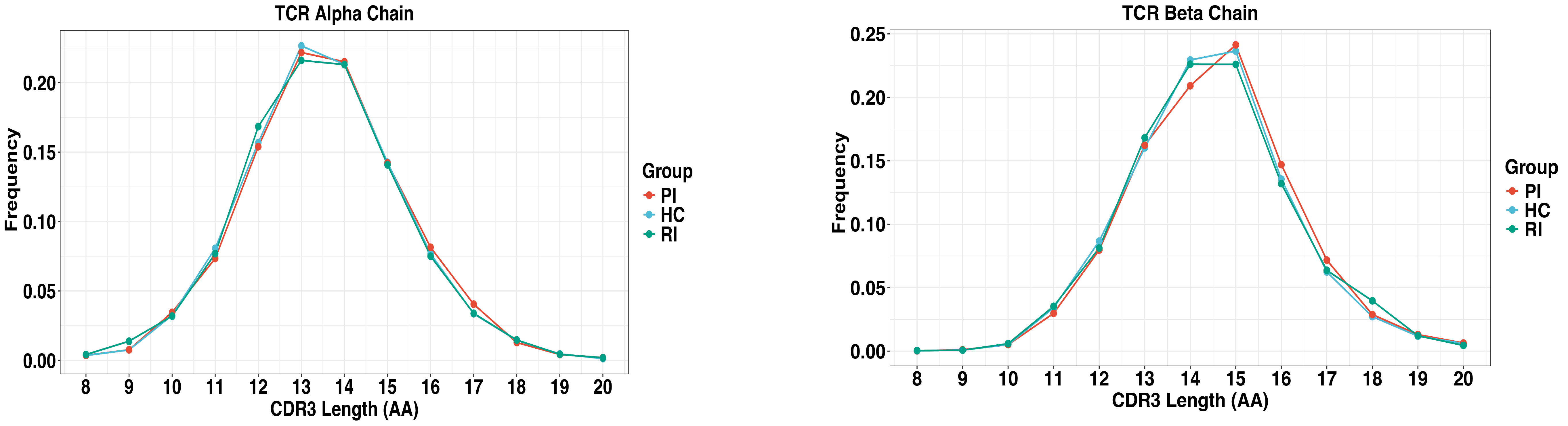


**Figure 8 A**mino acid (AA) length distributions of CDR3α and CDR3β in our experimentally detected TCR repertoires

CDR3β

CDR3α


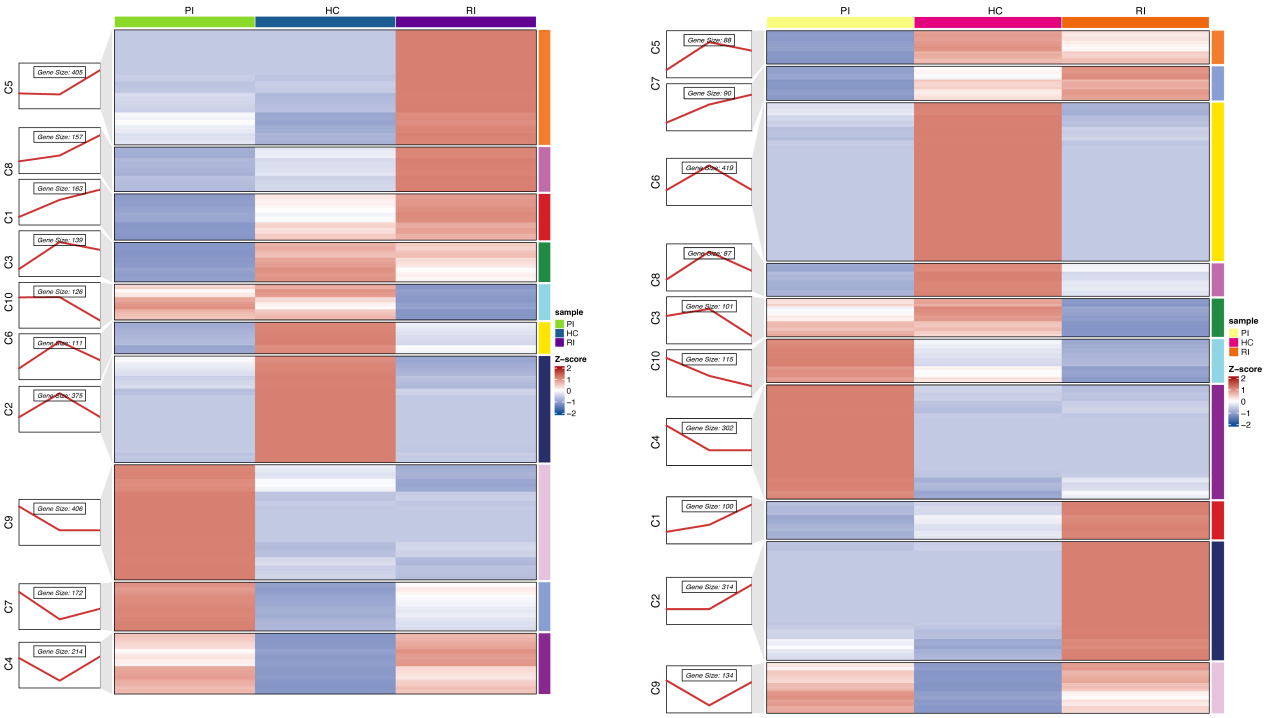


**Figure 9** Clustering analysis of COVID-19-linked CDR3αβ TCR sequences grouped by temporal frequency patterns via Mfuzz.


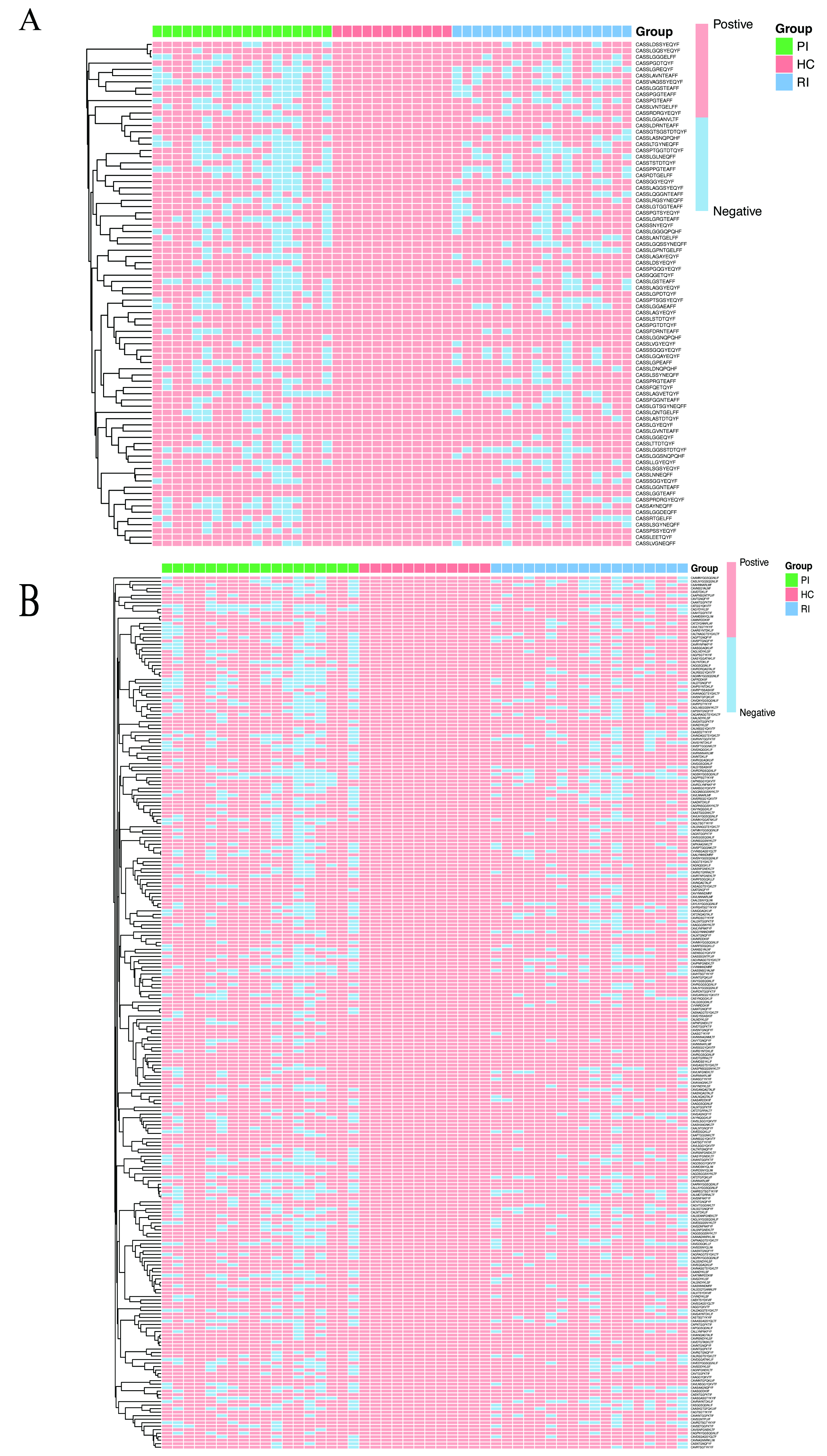


**Figure 10** Shared TCRα (A) and TCRβ (B) sequences among all HC patients.
